# Leaf shape in *Populus tremula* is a complex, omnigenic trait

**DOI:** 10.1002/ece3.6691

**Published:** 2020-10-13

**Authors:** Niklas Mähler, Bastian Schiffthaler, Kathryn M. Robinson, Barbara K. Terebieniec, Matej Vučak, Chanaka Mannapperuma, Mark E. S. Bailey, Stefan Jansson, Torgeir R. Hvidsten, Nathaniel R. Street

**Affiliations:** ^1^ Department of Plant Physiology Umeå Plant Science Centre Umeå University Umeå Sweden; ^2^ School of Life Sciences College of Medical, Veterinary and Life Sciences University of Glasgow Glasgow Scotland; ^3^ Faculty of Chemistry, Biotechnology and Food Science Norwegian University of Life Sciences Ås Norway

**Keywords:** complex trait, GWAS, leaf shape, natural variation, omnigenic, *Populus tremula*

## Abstract

Leaf shape is a defining feature of how we recognize and classify plant species. Although there is extensive variation in leaf shape within many species, few studies have disentangled the underlying genetic architecture. We characterized the genetic architecture of leaf shape variation in Eurasian aspen (*Populus tremula* L.) by performing genome‐wide association study (GWAS) for physiognomy traits. To ascertain the roles of identified GWAS candidate genes within the leaf development transcriptional program, we generated RNA‐Seq data that we used to perform gene co‐expression network analyses from a developmental series, which is publicly available within the PlantGenIE resource. We additionally used existing gene expression measurements across the population to analyze GWAS candidate genes in the context of a population‐wide co‐expression network and to identify genes that were differentially expressed between groups of individuals with contrasting leaf shapes. These data were integrated with expression GWAS (eQTL) results to define a set of candidate genes associated with leaf shape variation. Our results identified no clear adaptive link to leaf shape variation and indicate that leaf shape traits are genetically complex, likely determined by numerous small‐effect variations in gene expression. Genes associated with shape variation were peripheral within the population‐wide co‐expression network, were not highly connected within the leaf development co‐expression network, and exhibited signatures of relaxed selection. As such, our results are consistent with the omnigenic model.

## Graphical abstract

We characterized the genetic architecture of leaf shape variation in Eurasian aspen (*Populus tremula* L.) by performing a genome‐wide association studies (GWAS) for physiognomy traits. Our results identified no clear adaptive link to leaf shape variation and indicate that leaf shape traits are genetically complex, likely determined by numerous small‐effect variations in gene expression. Genes associated with shape variation were peripheral within the population‐wide co‐expression network, were not highly connected within the leaf development co‐expression network, and exhibited signatures of relaxed selection.

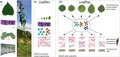

## 1 INTRODUCTION

Leaf shape is a defining feature of how we recognize and classify plant species and, as such, is an important component of our relationship with nature. Floral morphology and features are largely invariant within species and therefore served as the basis for taxonomic classification. In contrast, leaf shape varies distinctly between and, often extensively, within species. There are some identifiable global trends, indicating convergent evolution, such as a narrowing and more defined serration of leaves toward latitudinal extremes (Peppe et al., 2011; Royer, McElwain, Adams, & Wilf, 2008; Royer, Wilf, Janesko, Kowalski, & Dilcher, 2005; Traiser, Klotz, Uhl, & Mosbrugger, 2005). Despite the vast diversity of leaf shapes represented across plant species, our knowledge of the genetic architecture of natural variation in leaf shape, the evolutionary drivers, and adaptive significance of that variation or the molecular control of leaf development remains relatively limited (Chitwood & Sinha, 2016; Ichihashi et al., 2014).

Leaf organogenesis is initiated within an apical meristem from a group of dividing, undifferentiated initial (meristematic) cells (Clark, 1997). As the meristematic cells divide, the daughter cells reach the meristem peripheral zone and enter determinate growth. Once this initiation phase is complete, a boundary region between the meristem and the outgrowing lateral organ is established (Sluis & Hake, 2015), followed by the expansion of leaf primordia (Cleland, 2001). Leaf growth is a tightly coordinated process involving both cell proliferation (division) and expansion (Czesnick & Lenhard, 2015), both of which are coordinated by polar hormone distributions (Moon & Hake, 2011; Sluis & Hake, 2015). Leaf morphogenesis continues with growth in the proximo–distal, adaxial–abaxial, and medial–lateral axes, with polarized cell division and expansion along each axis creating a flat leaf lamina and with spatially varying rates of expansion and division determining basic leaf shape (Moon & Hake, 2011; Tsukaya, 2005).

A number of genes with a central role in the control of leaf primordia initiation and subsequent leaf development and pattern formation have been identified from forward genetic screens, largely in *Arabidopsis thaliana* (Tsukaya, 2005). For example, genes including the NAC transcription factors *NO APICAL MERISTEM* and *CUP‐SHAPED COTYLEDONS* (*CUC*) are expressed at the boundary region to delineate outgrowing leaf primordia from the meristem (Aida, Ishida, Fukaki, Fujisawa, & Tasaka, 1997; Cheng et al., 2012; Souer, van Houwelingen, Kloos, Mol, & Koes, 1996; Vroemen, Mordhorst, Albrecht, Kwaaitaal, & de Vries, 2003). Most classically, *ANGUSTIFOLIA* (*AN*) and *ROTUNDIFOLIA* (*ROT*) act independently to control polar length and width expansion, respectively (Tsuge, Tsukaya, & Uchimiya, 1996). There are also a number of notable examples of large‐effect genes underlying variation in leaf complexity (dissection), such as the *A. thaliana* HD‐zip transcription factor *LATE MERISTEM IDENTITY1 (LMI1)* homolog underlying leaf morphs in cotton (Andres et al., 2017).

Although forward genetic screens have identified genes acting during leaf organogenesis, most studies have been conducted using *A. thaliana* mutants that target single genes. Although those genes can be shown to be essential for, or to contribute to, the control of leaf development, they are not necessarily those underlying natural variation. Leaf shape is a complex, multigenic trait (Chitwood, Kumar, et al., 2013; Chitwood, Ranjan, Martinez, et al., 2014; Gupta, Rosenthal, Stinchcombe, & Baucom, 2019), meaning that causal variation in protein‐coding sequence or expression of each contributing gene will be of small‐effect size in relation to the total variation in the population. As such, alternative approaches are needed to identify loci underlying natural variation. One such approach is to use genetic screens to identify genomic regions involved in the control of complex traits. For example, genome‐wide association study (GWAS) or quantitative trait locus (QTL) mapping can be used, with the results being integrated with targeted expression studies or used to select candidates for forward genetic validation. A number of studies have used such integrative approaches to study leaf shape. Xiao et al. (2014) used a system genetic approach to study the genetic architecture of leaf development in a *Brassica rapa* double haploid population, identifying a cohort of candidate genes. Those candidates included well‐characterized examples such as *AINTEGUMENTA* (*ANT*), *CUC2,* and *GIBBERELLIN 20‐OXIDASE 3* (*GA20OX3*) in addition to numerous genes with no currently assigned function during leaf development. Tight genetic regulation of leaf traits has also been reported in *Vitis vinifera,* with grape leaves displaying large‐scale morphological variation among cultivars. Chitwood, Ranjan, Martinez, et al. (2014) and Gupta et al. (2019) showed that many leaf shape traits, including venation patterning, are highly heritable and that genes displaying differential expression can be identified between cultivars with contrasting leaf shapes. A number of studies of leaf shape have similarly been performed using tomato introgression lines and backcross inbred line populations of crosses between domesticated tomato (*Solanum lycopersicum*) and wild *Solanum* spp. (Chitwood, Kumar, et al., 2013; Chitwood, Ranjan, Kumar, et al., 2014; Fulop et al., 2016), including transcriptional studies (Chitwood, Maloof, & Sinha, 2013; Ichihashi et al., 2014; Ranjan et al., 2016). Phenotypic and expression GWAS in *A. thaliana* and transcription profiling in recombinant inbred lines of *Zea mays* have shown that the balance of cell division and expansion determining leaf growth differs among genetic backgrounds (Clauw et al., 2016).

*Populus* is an important model system for genomic, ecological, and evo‐devo studies. *Populus* species were appropriately described by Stettler and Bradshaw (1996) as being “replete with variation,” a statement that is particularly relevant to their extensive variation in leaf shape. Within the genus, the aspen species *P. tremula* and *P. tremuloides*, for example, contain extensive intraspecific natural variation in leaf shape (Barnes, 1969, 1975; Bylesjö et al., 2008), heterophylly along extending long shoots (Cox, 2005; Curtis & Lersten, 1978) and striking heteroblasty between leaves produced at the extending shoot apical meristem and those of short shoot buds (Critchfield, 1960). The pre‐formed leaves of most aspen species have flattened petioles that cause the characteristic trembling or quaking of the leaf lamina in very light wind. A number of QTL studies have been performed using hybrid *Populus* crosses, which have highlighted the polygenic control and heritability of leaf physiognomy traits (Drost et al., 2015; Lindtke, González‐Martínez, Macaya‐Sanz, & Lexer, 2013; Rae, Ferris, Tallis, & Taylor, 2006; Wu, Bradshaw, Stettler, & Stettler, 1997). Using a pseudo‐backcross pedigree of narrow‐leaf *P. trichocarpa* (section *Tacamahaca*) and broad‐leaved *P. deltoides* (section *Aigeiros*), Drost et al. (2015) identified a major QTL for leaf lamina width and length:width ratio. The mapped locus contains an ADP‐ribosylation factor (ARF), which is a strong candidate gene for the regulation of leaf morphology. Street et al. (2008), Street, Jansson, and Hvidsten (2011) performed system biology analyses to link available microarray expression data to leaf physiognomy QTLs, identifying *GRF*s (*growth‐regulating factors*) as candidate genes controlling leaf development. There are also a small number of reports of aberrant or altered leaf phenotypes resulting from genetic transformation studies (Du, Mansfield, & Groover, 2009; Ratke et al., 2018; Rottmann et al., 2000).

We used a GWAS of physiognomy traits in aspen to demonstrate the complex genetic architecture underlying the observed phenotypic variation. Existing population RNA sequencing (RNA‐Seq) gene expression data (Mähler et al., 2017) were used to identify genes differentially expressed between groups of genotypes with contrasting leaf shapes, and these were examined for the presence of SNPs associated with expression variation. To examine whether genes underlying natural variation in these complex traits were central within the developmental transcriptional program, we generated an RNA‐Seq developmental series of terminal leaves and used this to infer an aggregate co‐expression network. We examined the developmental profiles of GWAS candidate genes and explored their co‐expression network centrality. Furthermore, we used the population gene co‐expression network to characterize centrality of candidate genes associated with shape variation and to investigate signatures of selection. Taken together, our results indicate that leaf shape variation in aspen is highly polygenic and is associated with numerous small‐effect size variants affecting genes expressed during leaf development.

## 2 MATERIALS AND METHODS

### 2.1 Leaf shape phenotyping in the Swedish Aspen collection

Leaf size and shape parameters were measured in a natural population of *Populus tremula*, the Swedish Aspen (SwAsp) collection, growing in common gardens at Sävar, northern Sweden (63.9°N, 20.5°E), and Ekebo, southern Sweden (55.9°N, 13.1°E). The common garden trials comprised of natural (wild‐growing) aspen genotypes collected in 2003 across ten latitudinal degrees, which were cloned and planted in 2004 in a randomized block design in each garden (Luquez et al., 2008). Leaf samples were harvested in Sävar on 14 July 2008 and 28 June 2011 and in Ekebo on 18 July 2008 and 4 August 2011, when leaves were fully expanded and mature, but before the occurrence of substantial damage due to herbivory or the presence of fungal rust infection. Ten undamaged leaves per replicate tree were sampled randomly across the canopy, avoiding leaves from the first or last leaf in a leaf cohort originating from a single bud. In total, 430, 444, 326, and 393 trees were sampled in Ekebo 2008, Ekebo 2011, Sävar 2008, and Sävar 2011, respectively, comprising between 1 and 8 (median = 3) clonal replicates. One hundred and thirteen genotypes were sampled in both years in Ekebo and in 2011 in Sävar, and 111 genotypes were sampled in 2008 in Sävar. Leaves were stored at 4–8°C immediately after harvest. Petioles were removed at the leaf base, and the sample of ten leaves per tree was scanned in color at 300 dpi using a CanoScan 4400F. A 5 × 4 cm Post‐it note was scanned as a scale image. The resulting images were analyzed using LAMINA (Bylesjö et al., 2008) to obtain leaf size and shape metrics. Since LAMINA quantifies 25 leaf shape and size metrics, many of which are highly correlated, we selected leaf area (a size trait), circularity (a shape trait), and indent depth (a trait with elements of both size and shape) as representative phenotypes for this study. Median values of the ten leaves per tree were calculated for each leaf size and shape metric, and the median value per individual was used for all subsequent analyses, including genetic correlations, GxE analyses, heritability, and Q_ST_.

### 2.2 Statistical analyses

All statistical analyses of the SwAsp collection physiognomy data were conducted in R. Phenotypic data were examined for homogeneity of variance. No data transformations were required to meet the assumptions of a normal distribution. Pearson's correlations were used for all phenotypic correlations calculated.

Estimates of broad‐sense heritability (*H* ^2^) and their 95% confidence intervals, including all clonal replicates, were calculated as

$$H^{2}=V_{G}/\left(V_{G}+V_{E}\right)$$

using median values of ten leaves per clonal replicate, where *V*_G_ and *V*_E_ are genetic and environmental variance components, using the heritability function in the R package “Heritability.” To estimate population differentiation, *Q*_ST_, the following formula was used:

$$Q_{\text{ST}}=V_{\text{pop}}/\left(V_{\text{pop}}+\left(2*V_{\text{geno}}\right)\right)$$

where *V*_pop_ and *V*_geno_ are the interpopulation and genotype (*i.e.,* interindividual) genetic variance components, respectively.

Genetic variances (*V*_G_) were calculated using linear mixed models in the nlme package in R (Pinheiro, Bates, DebRoy, Sarkar, & R Core Team, 2020) specified as

y∼1,random=∼1|genotype/plantID

where *y* is the phenotype, the median of 10 leaves per tree or the sum of the two traits for which covariance is calculated, genotype is the genotype of the sampled tree modeled as a random effect, and plantID is the plant replicate nested within genotype. The genetic variance components (V_G_) were estimated from each linear mixed model object using the varcomp function in the ape package in R (Paradis & Schliep, 2019). Genetic correlations were then estimated between each pair of phenotypes, where phenotypic values were medians of ten leaves per tree, as

$$r_{G(\text{AB})}=V_{G(\text{AB})}/\sqrt{\left(V_{G(A)}\times V_{G(B)}\right)}$$

where *r*_G(AB)_, the genetic correlation of phenotype A and phenotype B, was calculated from the *V*_G(AB)_, the genetic covariance in phenotype A and phenotype B, and *V*_G(A)_ and *V*_G(B)_ were the genetic variances of phenotypes A and B, respectively.

Genetic (clonal) variation for each phenotype between years and common gardens was investigated using analysis of variance (ANOVA) using the following model specified in R as

y∼site+year+site:year

where the phenotype *y* was the median of 10 leaves per tree, range normalized from 0 to 1 for each phenotype to facilitate comparison of *F*‐value magnitudes among traits. The R package scales (Wickham & Seidel, 2020) were used for range normalization. The terms site and year were the independent variables for measurement year and common garden, respectively. ANOVA models were implemented in the aov function in R. All effects were considered significant at *p* < .05.

### 2.3 Swedish Aspen collection RNA sequencing data (LeafPop dataset)

The RNA‐Seq data used in this study were described in Mähler et al. (2017). We refer to this dataset here as LeafPop. It comprises 219 samples distributed among 86 distinct genotypes. The samples were leaf buds collected at an early, defined point of bud flush. As such, the data represent a single snapshot during leaf development of pre‐formed leaves. The same type of gene expression filtering and adjustment was used in this paper as in Mähler et al. (2017). Genes were required to have an expression variance above 0.05, and the first nine gene expression principal components were regressed out from the data. This left 22,306 genes for further analysis. The data are available at the European Nucleotide Archive (ENA) as accession ERP014886.

### 2.4 Genome‐wide association mapping

A total of 4.5 million SNPs were considered for the GWAS, as detailed in Mähler et al. (2017). Best linear unbiased predictors (BLUPs) of the three leaf traits considered (leaf area, indent depth, and circularity) were calculated using the lmer function in the lme4 R package. The model used was specified in R as

y∼garden+year+block+(1|genotype)

where *y* is the phenotype, garden is the common garden where the phenotype was measured, year is the year in which the phenotype was measured, block is the location of the tree in the common garden, and genotype is the genotype of the sampled tree.

For GWAS of traits in individual gardens and years, BLUPs were similarly calculated as

$$y∼\text{block}+\left(1|\text{genotype}\right)$$

where *y* is the phenotype measured, block is the location of the tree in the common garden, and genotype is the genotype of the sampled tree.

A univariate linear mixed model was applied to the data using GEMMA v0.94 (Zhou & Stephens, 2014) and included days to bud set (Wang et al., 2018) as a covariate in order to account for any latent variation due to latitudinal population differentiation in the phenotype, as well as the built‐in estimation of a centered relatedness matrix to control for population structure and relatedness. GEMMA produces different statistics for significance, and we used *p*‐values based on a likelihood ratio test. These *p*‐values were consequently Benjamini–Hochberg‐adjusted for multiple testing for each garden and year separately using the p.adjust function in R. To estimate the proportion of variance explained (PVE) by all SNPs for each phenotype, a Bayesian sparse linear mixed model (BSLMM) was applied in GEMMA using the Markov chain Monte Carlo (MCMC) method (Zhou, Carbonetto, & Stephens, 2013). For each of the three traits, MCMC chain length was set to 1,000,000 steps with the first 250,000 discarded as burn‐in and thinned to every 100th sample resulting in 10,000 independent draws from the posterior distribution. The median of the posterior distribution of the pve parameter was taken as a point estimate of the proportion of the phenotypic variance explained by all SNPs. To associate genes with SNPs, the v1.0 *Populus tremula* annotations from the PopGenIE.org web resource (Sundell et al., 2015) were used, and any gene within 2 Kbp of a SNP was said to be associated with that SNP.

The top 1,000 genes for each of the three traits were selected by rank ordering the associations by significance and then walking down the list until 1,000 unique genes had been selected.

### 2.5 GWAS gene set enrichment analysis

The gene set enrichment analysis performed was inspired by Subramanian et al. (2005). The most significant SNP within 2 Kbp of a gene was used to rank all genes found in the GWAS results. We observed a weak negative relationship between gene length and the minimum *p*‐value (*r* ^2^ = 0.06), which we considered small enough to not account for. For each gene set, a running sum was made where the value was increased proportionally to the *p*‐value of the most significant association within the gene, if the gene was in the gene set being tested, otherwise the value was decreased. The maximum value in this running sum acted as a test statistic, and 10,000 permutations of the gene ranking were performed, and empirical *p*‐values were calculated. The genes in the gene set tested that contributed to the maximum score in the running sum, that is, genes that occurred before or at the maximum in the running sum, were considered part of the leading‐edge subset. The algorithm was implemented in C++ using Rcpp (Eddelbuettel & Balamuta, 2017) and is available at https://gist.github.com/maehler/239e18e7d9f2b53c792c05f2aca5cebd. Multiple testing correction was performed using the qvalue function in the qvalue R package. Only gene sets containing more than five genes were considered for the enrichment test. The exponent parameter of the GSEA test was set to 1 for all tests.

### 2.6 Genotype extreme group differential expression

The top and bottom quartiles of the BLUPs for leaf area, indent depth, and circularity were contrasted in the gene expression data from the SwAsp population (Mähler et al., 2017) using DESeq2 (Love, Huber, & Anders, 2014). All data were used as input, and only after the model had been created, the top and bottom quartiles were contrasted. This was done in order to maintain information from samples with intermediate phenotypic values for the dispersion estimation. Genes with an adjusted *p*‐value <0.05 were considered differentially expressed. Functional enrichment tests were performed as detailed above.

When testing for differences in network centrality among differentially expressed genes, a randomization scheme was employed in order to assert that there was no circular reasoning behind the connection between DE and network centrality. The sample labels within the top and bottom quartiles for leaf area were shuffled, and differential expression analysis was performed on this new dataset. This was repeated 100 times for leaf area in order to get an indication as to whether DE between random subsets of samples was inherently connected with network centrality.

### 2.7 Plant Material (LeafDev dataset)

We collected root cuttings of diameter 5–10 mm from a wild‐growing clonal stand of *Populus tremula* in northern Sweden (as detailed in Sundell et al., 2017) on 29 July 2013. We divided the root cuttings into 25 cm lengths and placed these onto prewatered potting compost (K‐jord) in trays and then covered the root sections with 2 cm of additional compost. The trays were kept damp and placed in a greenhouse with mean day/night temperature of 20/15°C, humidity 50%–70%, and an 18‐hr photoperiod. After three weeks, vegetative shoots of approximately 5 cm height were separated from the root sections and planted into two‐liter pots containing potting compost (K‐jord) and grown in a greenhouse for 12 weeks (24‐hr light, 22 degrees, 50%–70% humidity).

Leaf Plastochron Index (LPI; (Erickson & Michelini, 1957; Larson & Isebrands, 1971; Meicenheimer, 2014) has been extensively used for *Populus* research as a means to sample leaves of equivalent developmental age from replicate plants. We first established that the production of terminal leaves in aspen obeys the assumptions of LPI.

We collected a developmental series of terminal leaves to perform morphological and transcriptional assays. The first fully unfurled leaf was defined as a reference point and was labeled leaf T0. Three leaves above the reference leaf (labeled as T‐1, T‐2, and T‐3) and the apical region, containing the shoot apical meristem and the very youngest leaf primordia (labeled T‐4), and two leaves below the reference leaf (labeled T1 and T2) were sampled from five replicate trees for RNA extraction and from an additional five replicate trees for morphological analyses. Samples collected for RNA extraction were immediately flash‐frozen in liquid nitrogen and stored at −80°C.

### 2.8 RNA extraction

We extracted total RNA from terminal leaves above the reference leaf (samples T‐1 through T‐4) using the RNAqueous Micro Kit (Life Technologies) according to the manufacturer's guidelines. For all other terminal leaves (samples T0 through T2), RNA was isolated using the mirVana Kit (Life Technologies) according to the manufacturer's guidelines. DNA was removed using the DNA‐free™ DNA Removal Kit (Life Technologies) according to the manufacturer's instructions. RNA purity was measured using a NanoDrop 2000 (Thermo Scientific) and RNA integrity assessed with a Plant RNA Nano Kit using a Bioanalyzer 2000 (Agilent Technologies) using the plant total RNA setting.

### 2.9 Leaf physiognomy

To enable analysis of leaf size and shape (physiognomy) parameters, we sampled a terminal leaf developmental series for which a reference leaf was defined as above. We sampled one leaf younger than the reference leaf (leaf T‐1), the reference leaf itself (leaf T0), and five subsequent leaves older than the reference leaf (leaf T1 to leaf T5). Leaves younger than leaf T‐1 were not suitable for use in this analysis. As such, the RNA set of samples profiled leaves younger than were represented in this physiognomy developmental series. This developmental series was sampled from five clonally replicated trees.

To obtain images for all leaves, we first removed the petioles and subsequently scanned the leaves on a flatbed scanner (Canon LiDE210). Scanned images were saved as 300‐dpi color JPEG files. Leaf shape parameters were then calculated using LAMINA (Bylesjö et al., 2008). Quantified traits included leaf area, length, width, serration number, and dimensions in addition to a number of calculated traits such as aspect ratio (length:width) and circularity.

### 2.10 RNA sequencing data preprocessing

RNA‐Seq of total RNA was performed by the Beijing Genome Institute using the Illumina sequencing platform with mRNA assayed using >20 million 2 × 100 bp paired‐end reads per sample. The data are deposited in the European Nucleotide Archive (ENA) as accession PRJEB31491. Protocol details were as presented in Nystedt et al. (2013).

An in‐house pipeline that combines a number of existing tools was used for data processing and expression value calculation (Delhomme et al., 2015). Sequence data quality was assessed using FastQC/0.10.1 (Andrews,n.d.). Sequence reads originating from ribosomal RNAs (rRNA) were identified and removed using SortMeRNA/1.9 (Kopylova, Noé, & Touzet, 2012) for which libraries rfam‐5s, rfam‐5.8s, silva‐arc‐16s, silva‐bac‐16s, silva‐euk‐18s, silva‐arc‐23s, silva‐bac‐23s, and silva‐euk‐28s were used. The sequence data were then processed to remove low‐quality bases or entire reads, and adapter contamination was removed using Trimmomatic/0.32 (Bolger, Lohse, & Usadel, 2014). The trimming parameters used were SLIDINGWINDOW:5:20 MINLEN:50 while trimming the adapter TruSeq3‐PE. After each of rRNA removal and quality filtering, the remaining sequence data were assessed again using FastQC/0.10.1. The reads were mapped to the de novo *Populus tremula* v1.0 genome (available at PopGenIE.org) using STAR/2.4.0f1 (Dobin et al., 2013) with the settings –runThreadN 16, ‐‐readFilesCommand zcat, ‐‐limitBAMsortRAM, ‐‐outQSconversionAdd ‐31, ‐‐outSAMtype BAM SortedByCoordinate, ‐‐outSAMstrandField intronMotif, ‐‐outSAMmapqUnique 254, ‐‐outWigType bedGraph, ‐‐outFilterMultimapNmax 100, ‐‐alignIntronMax 11000, ‐‐chimSegmentMin 1, ‐‐sjdbGTFfile Potra01‐gene‐wo‐intron.gtf ‐‐quantMode TranscriptomeSAM. Alignments to features were counted to enable gene loci expression quantification using HTSeq/0.6.1 (Anders, Pyl, & Huber, 2014), for which a GFF3 file containing a representative transcript per coding loci was used.

### 2.11 Differential expression inference and functional enrichment analysis

The gene expression data were first visually assessed by performing a principal component analysis (PCA) and clustered heat maps using blind variance stabilized data (Lin, Du, Huber, & Kibbe, 2008). This identified one sample of leaf 4 from the terminal leaf development series as a clear outlier. We therefore excluded this sample from subsequent analysis. Differentially expressed genes (DEGs) between developmental stages were inferred within the R framework (R Core Team, 2018) using DESeq2 (Love et al., 2014) with a formula including the replicate and developmental stage as a term (~ Replicate + Time).

Gene Ontology (Ashburner et al., 2000) functional enrichment (over‐representation) of DEGs at *p* < 0.05 was analyzed using an in‐house implementation of the parent–child test (Grossmann, Bauer, Robinson, & Vingron, 2007), and PFAM domain (Finn et al., 2010) enrichments were calculated using a hypergeometric test.

### 2.12 Gene network inference

We inferred a gene co‐expression network for the terminal leaf RNA‐Seq dataset. The expression data were first filtered to only include genes with nonzero expression in at least $\left[\sqrt{N}\right]$ samples, which were transformed to homoscedastic, asymptotically log2 counts using the regularized log transformation as implemented in DESeq2. Ten network inference methods were computed using the Seidr 0.9 toolkit (Schiffthaler, Serrano, Delhomme, & Street, 2018)—ARACNE, CLR, GENIE3, Narromi, PCor, Pearson, PLSNET, Spearman, SVM, and Tigress. For each symmetric edge pair, the one with the higher score was kept in case of nonsymmetrical scoring by the algorithm. The networks were aggregated using the inverse rank product method (Zhong, Allen, Xiao, & Xie, 2014), and edges were filtered according to the noise corrected backbone (Coscia & Neffke, 2017) at a sigma of 2.32 (which roughly corresponds to a *p*‐value of 1%). Network partitions were identified using InfoMap (Rosvall & Bergstrom, 2008) with default settings. Graph layout and images were created using Gephi 0.9.2. Node centrality statistics were calculated using Seidr 0.9, except for Kleinberg's Hub and Authority, which was calculated using Gephi 0.9.2.

## 3 RESULTS

**FIGURE 1:**
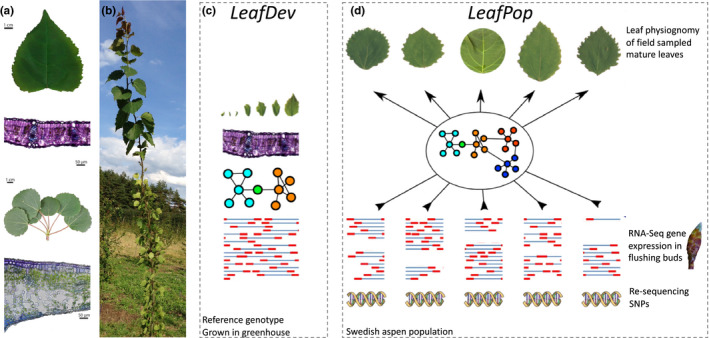
*Populus tremula* leaf physiology and morphology. (a) Representative mature terminal leaf (top) and flushed short shoot bud with mature pre‐formed leaves (bottom). Corresponding cross sections of a representative mature terminal leaf (15 µm thick) and mature pre‐formed leaf (10 µm thick) are shown. Cross sections were embedded in 1.5% plant agarose, sectioned using a vibratome, and stained for one minute with toluidine blue; magnification 200×. (b) Example field‐grown tree displaying clear heteroblasty with terminal leaves toward the top of the stem and pre‐formed leaves on the lower half of the stem. (c) A reference genotype was clonally replicated and grown in the greenhouse to generate a developmental sample series of terminal leaves. This dataset is referred to as LeafDev and is used to provide developmental context to candidate genes and to generate a developmental co‐expression network. (d) The Swedish Aspen population was used to sample replicated mature pre‐formed leaves to perform GWAS for leaf physiognomy traits. Gene expression was assayed from flushing buds to provide a developmental snapshot of expression variation among genotypes. This dataset is referred to as LeafPop

We first performed a characterization of the two heteroblastic leaf forms produced by *P. tremula*. Pre‐formed leaves were orbicular and those produced from the extending shoot apical meristem (terminal) were cordate (Figure 1a,b), which is in agreement with previous observations in poplar (Liu et al., 2015; Russin & Evert, 1984). Pre‐formed leaves were notably thicker (~2×) than terminal leaves, with contrasting spatial arrangement of cell layers between the two leaf types (Figure 1a). Pre‐formed leaves had a thicker adaxial epidermal layer followed by two rows of densely packed palisade mesophyll; spongy mesophyll cells were separated by many air spaces and a thick abaxial epidermis. The spongy mesophyll cell layer of terminal leaves had fewer air spaces than in pre‐formed leaves (Figure 1a).

The following analyses comprise population‐wide phenotype and gene expression (LeafPop) data integrated with developmental gene expression data (LeafDev) generated from a single reference genotype (Figure 1c,d).

### 3.1 Genome‐wide association mapping identifies complex genetic architecture

**FIGURE 2:**
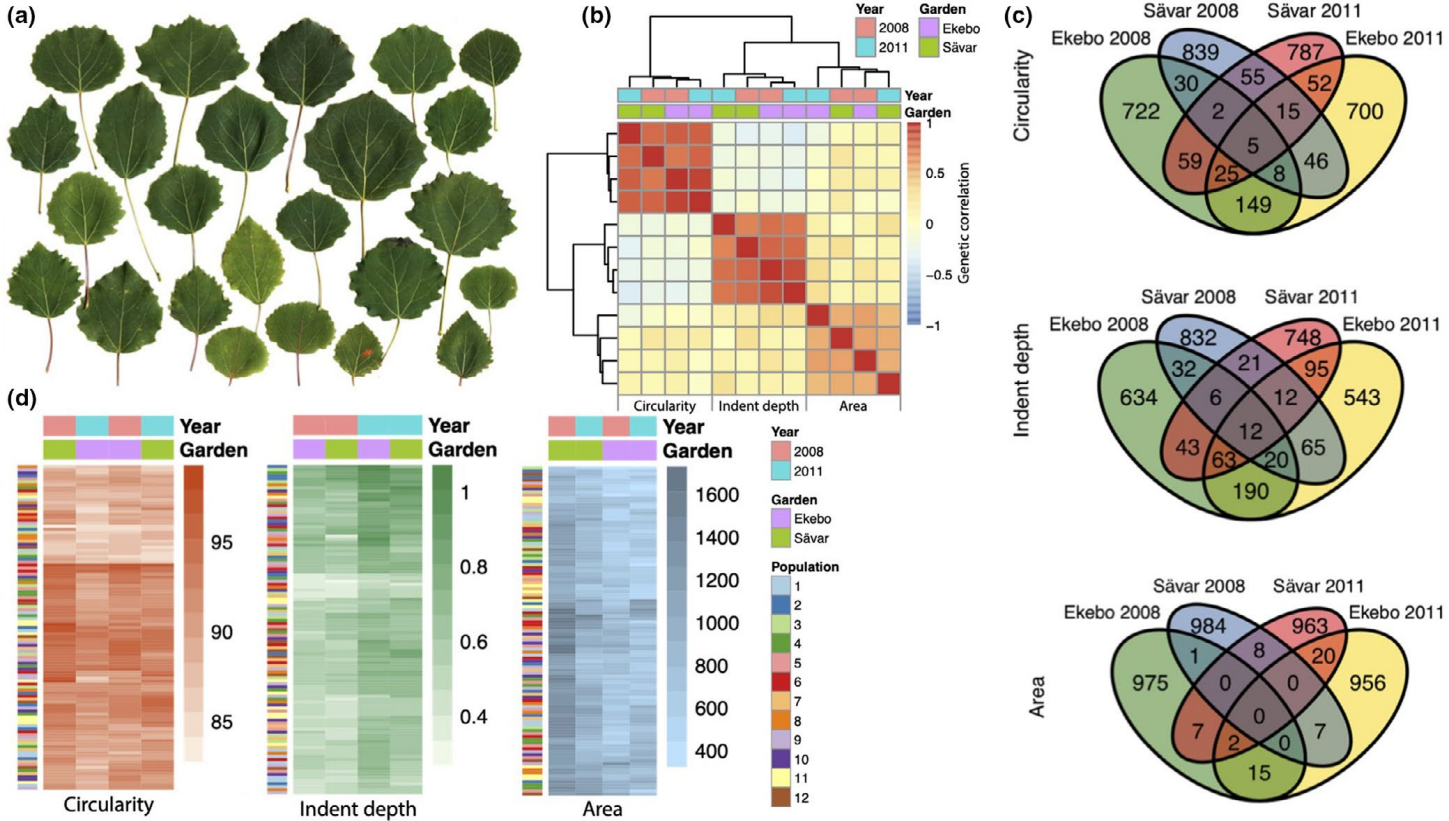
An overview of leaf circularity, indent depth, and leaf area in the Swedish Aspen (SwAsp) collection. (a) Example leaves from the SwAsp collection. Each leaf is from one genotype, and the set of leaves was selected to reflect the extent of variation represented among all genotypes. (b) Genetic correlations among measures of leaf circularity, median indent depth, and leaf area for data recorded in two common gardens (Ekebo, southern Sweden, and Sävar, northern Sweden) and two years (2008 and 2011). (c) Intersection of the top 1,000 ranked SNPs from genome‐wide association mapping for the three traits measured in the two years and gardens. (d) Heat map representations of leaf circularity (left), median indent depth (center), and leaf area (right) in the two common gardens and years. Each represented value is a median calculated from 10 leaves per clonal replicate (median *n* = 3) of each genotype. In each heat map, genotypes are clustered by Euclidean distance with the population from which the genotype originates indicated by colored bars to the left of the heat map. The values represented were used to perform the genome‐wide association mapping results depicted in “c”

We characterized variation in pre‐formed leaf shape within the Swedish Aspen (SwAsp) collection of *P. tremula* genotypes, which was sampled from local populations in Sweden and established in two replicated common garden experiments located in the north (Sävar, Umeå, 62°N) and south (Ekebo, 56°N) of Sweden (Luquez et al., 2008). We measured three representative leaf physiognomy traits: leaf area, circularity, and indent depth (margin/boundary serration) in both common gardens in two years. We observed extensive natural variation in pre‐formed leaf shape (Figure 2a), with measured leaf shape traits having high broad‐sense heritability (*H* ^2^) and low subpopulation differentiation (*Q*_ST_) (Table 1). Genetic correlations indicated that a substantial proportion of the heritable variation for each trait is controlled by genetic factors unique to that trait (Figure 2b; Table 1). We additionally tested whether any of the traits correlated significantly to a range of environmental and climatic factors (Table 1). There was no evidence of subpopulation differentiation for any of the traits, as indicated by low *Q*_ST_ (Table 1), and genotypes did not cluster by subpopulation of origin on the basis of the three phenotypic traits (Figure 2d). Similarly, there were no large‐effect significant correlations between the traits and environmental and climatic factors (Table 1). Leaf circularity and indent depth had higher *H* ^2^ and lower genotype‐by‐environment (GxE) interaction than did leaf area (indicated by comparisons of ANOVA *F* values; Table 1; Figure S1).

**TABLE 1:** Broad‐sense heritability (*H* ^2^), population differentiation (*Q*_ST_), genotype‐by‐environment interaction (G × E), and environmental correlations for leaf circularity, indent depth, and area. Phenotypes were measured in two years (2008 and 2011) and two common gardens (Ekebo in southern Sweden and Sävar in northern Sweden). G × E interactions for each phenotype were tested using an ANOVA model with garden, year, and garden × year as independent variables, and phenotype as the dependent variable. The *F* ratio and *p*‐value for the garden:year interaction are reported. Figure S1 details comparisons for each year/garden combination

	Garden/year	Circularity	Indent depth	Area
H^2^ (95% C.I.)	Ekebo 2008	0.71 (0.64–0.78)	0.60 (0.51–0.69)	0.40 (0.30–0.51)
	Ekebo 2011	0.65 (0.56–0.72)	0.72 (0.65–0.79)	0.61 (0.52–0.69)
	Sävar 2008	0.75 (0.66–0.82)	0.62 (0.51–0.72)	0.24 (0.12–0.38)
	Sävar 2011	0.71 (0.63–0.78)	0.64 (0.55–0.73)	0.27 (0.16–0.29)
Q_ST_ (95% C.I.)	Ekebo 2008	0.16 (0.07–0.37)	0.03 (0.00–0.12)	0.09 (0.03–0.25)
	Ekebo 2011	0.04 (0.01–0.15)	0.02 (0.02–0.20)	0.12 (0.05–0.31)
	Sävar 2008	0.20 (0.09–0.45)	0.09 (0.03–0.28)	0.08 (0.02–0.25)
	Sävar 2011	0.11 (0.04–0.30)	0.05 (0.01–0.18)	0.06 (0.01–0.20)
GxE ANOVA (F)		5.309	36.18	231.0
GxE ANOVA (*p*)		0.0213	<2.23e‐09	<2e‐16
Latitude R^2^	Ekebo 2008	0.031	0.031	0.059
	Ekebo 2011	−0.008	0.034	0.069
	Sävar 2008	−0.006	0.051	0.006
	Sävar 2011	0.036	−0.004	−0.002
Longitude R^2^	Ekebo 2008	0.021	−0.009	0.009
	Ekebo 2011	−0.009	−0.002	0.017
	Sävar 2008	−0.008	−0.002	−0.001
	Sävar 2011	0.018	0.004	0.001
Elevation R^2^	Ekebo 2008	−0.007	0.017	0.010
	Ekebo 2011	−0.009	0.049	0.046
	Sävar 2008	0.008	0.006	−0.006
	Sävar 2011	0.008	−0.007	−0.009
Precipitation R^2^	Ekebo 2008	0.024	−0.008	0.057
	Ekebo 2011	−0.007	−0.008	0.011
	Sävar 2008	0.017	−0.009	−0.009
	Sävar 2011	0.031	0.018	−0.008

We used the trait values to perform association mapping using genome‐wide single nucleotide polymorphism (SNP) markers identified from population‐wide resequencing data (Wang et al., 2018). Since there were no statistically significant associations after multiple testing correction for any trait in any of the repeated datasets, we examined whether there was overlap among the top 1,000 SNP associations (rank‐ordered by *p*‐value), indicative of consistency in ranking among the repeated measures. In general, there were few associations in common among the repeated measures, with the majority of the top‐ranked associations being unique to each dataset (Figure 2c). In agreement with the lower GxE and higher *H* ^2^ for leaf circularity and indent depth, these traits displayed greater overlap among the associations than for leaf area. We also calculated best linear unbiased predictions (BLUPs) from the repeated measures of each trait, but again found no statistically significant associations after multiple testing correction for the BLUPs (Figure S2, Table S1). There was no evidence of substantial inflation due to population stratification (Yang et al., 2011), as indicated by genomic control (GC) values (λGC 1.05 for area, 1.04 for circularity, 1.04 for indent depth), while SNP‐based estimates of percentage variance explained (PVE) were relatively high (0.40 ± *SD* 0.31 for area, 0.63 ± *SD* 0.31 for indent depth, 0.80 ± *SD* 0.28 for circularity), further supporting higher plasticity of leaf area and that trait variance was under genetic control.

**FIGURE 3:**
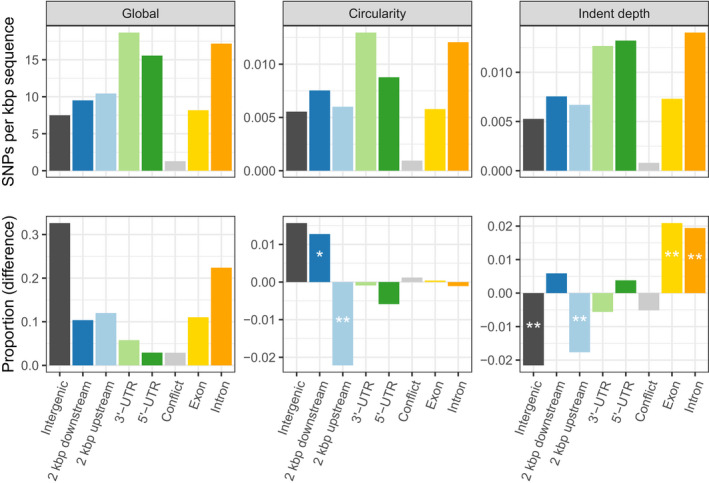
The genomic context distribution of SNPs within the top‐ranked 1,000 genes identified using association mapping for leaf circularity and indent depth. The upper panels show the SNP counts normalized by the total length of the features, genome wide. The bottom left panel shows the proportion of SNPs in the different features, while the bottom center and right panels show the difference in proportions among the top 1,000 genes compared with genome wide. Asterisks represents the significance of a proportion test (**p < 0.01; *p < 0.05). The "Conflict" feature represents overlapping 5' and 3' UTRs

To select candidate genes for these traits, we again rank‐ordered the SNP associations by *p*‐value and selected the top 1,000 genes for each of the traits (Table S2). The majority of associations were unique to each trait (Figure S3), in line with genetic correlations suggesting largely independent genetic control (Figure 2b). We examined the genomic context of SNPs within the top 1,000 gene sets (Figure 3), observing that the highest density of SNPs occurred in regulatory regions (UTRs and flanking regions, which contain the promoter).

### 3.2 Differential gene expression between genotypes with contrasting leaf shape

**FIGURE 4:**
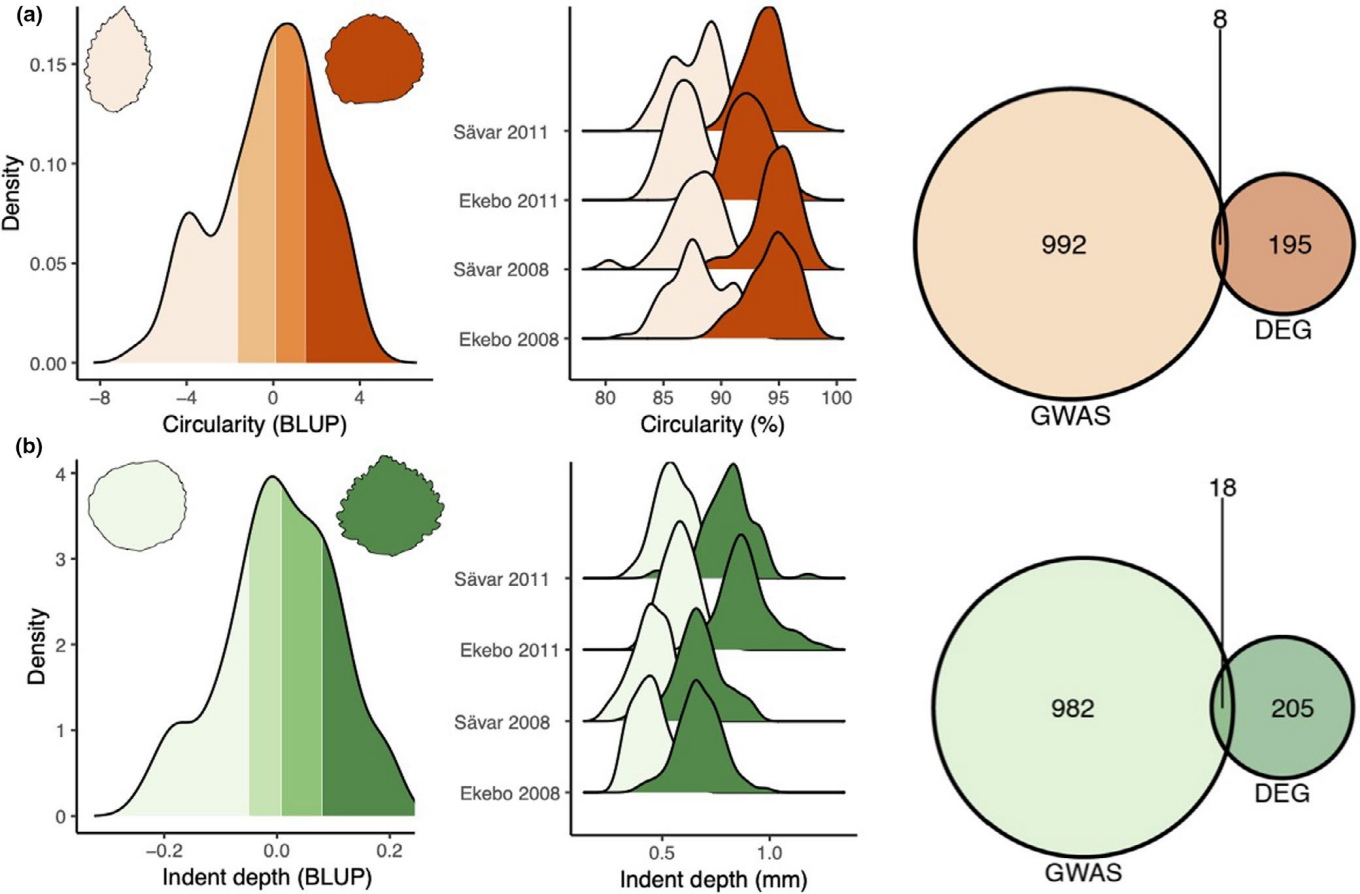
Selection of a set of high and low phenotype value genotypes. Genotypes with high and low phenotypic values of circularity (a) and indent depth (b) were selected on the basis of the top or bottom quartiles of the best linear unbiased prediction (BLUP) values for each trait. The color intensity reflects the four quartiles of trait values with darker colors meaning higher values. The left density plots represent BLUPs for the two traits. The distribution plots in the center depict the original trait measurements in the two gardens and the two years of sampling prior to the BLUP calculation. Gene expression assayed in young leaf buds (Mähler et al., 2017) was used to test for differential expression between genotypes within the low and high sets for each phenotypic trait. The Venn diagram shows the intersection between the significantly differentially expressed genes and the top 1,000 genes from the genome‐wide association study (GWAS) for the two traits

We used an existing resource assaying gene expression in flushing leaf buds from the SwAsp collection (Mähler et al., 2017) to examine correlations between gene expression and the leaf physiognomy traits. We refer to this dataset as LeafPop. Although none of these correlations were significant (Figure S4a), we did find significantly differentially expressed genes (DEGs) between sets of genotypes at the population distribution extremes for the three phenotypic measures (Figure 4) including 182 DEGs for area, 203 for circularity, and 223 for indent depth (Table S3).

### 3.3 Extensive remodulation of the transcriptome during leaf development

**FIGURE 5:**
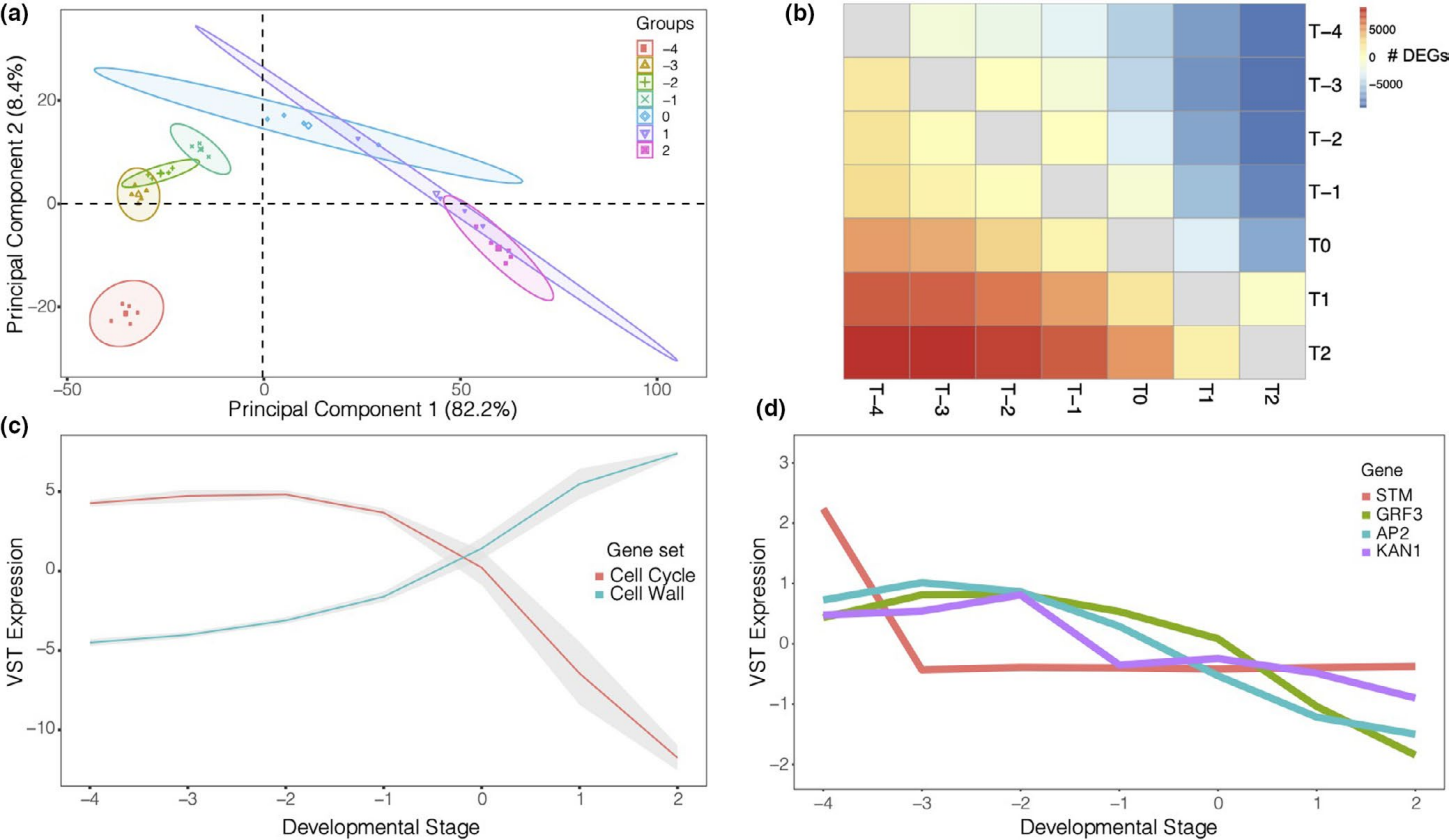
Overview of gene expression in developmental series of terminal leaves. (a) Principal component analysis of the 1,000 most variable genes. Data points are shaded by tissue type (Pf = pre‐formed; T = terminal) and leaf number in addition to different point styles being used. Ellipses around each group indicate a 95% confidence area for a particular sample group. (b) Differential expression matrix of all sample points showing all significantly upregulated genes (*p*_adj_ < 0.05) in the lower triangular matrix and all significantly downregulated genes (*p*_adj_ < 0.05) in the upper triangular matrix. In the lower triangular, columns constitute the numerator of the differential test, while rows constitute the numerator in the upper triangular half. T = terminal. (c) Eigengene expression of the Gene Ontology terms GO:0007049 (“Cell Cycle”) and GO:0009664 (“Cell Wall”) in the LeafDev dataset. The lines represent the mean eigengene (i.e., the first principal component) of all genes within each GO category. The gray ribbon indicates one standard error. (d) Mean expression of *Populus tremula* homologs (defined by best BLASTp) of *A. thaliana* genes of known function during leaf development. STM = *shoot meristemless* (Potra000526g03598/AT1G62360); GRF3 = *growth‐regulating factor 5* (Potra000751g05914/AT3G13960); AP2 = *Apetala 2* (Potra001894g15070/AT4G36920); KAN1 = *KANADI 1* (Potra008706g26216/AT5G16560)

**FIGURE 6:**
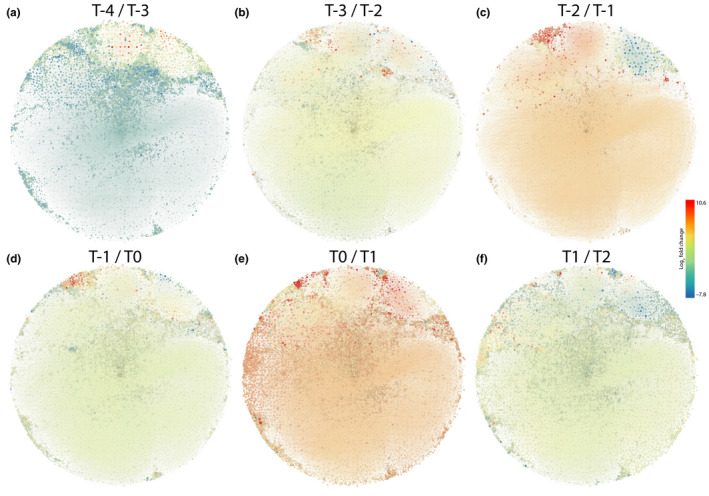
Visualization of differential expression within the terminal developmental series gene co‐expression network. (a) Transition between stages −4 and −3. (b) Transition between stages −3 and −2. (c) Transition between stages −2 and −1. (d) Transition between stages −1 and 0. (e) Transition between stages 0 and 1. (f) Transition between stages 1 and 2. Nodes are sized inversely by adjusted *p*‐value of a differential expression comparison at the relevant transition and shaded by the fold change (higher in red, lower in blue) with the earlier leaf number as the numerator. *p‐*values ranged from 1.519197e‐104 to 9.999962e‐01 with the highest *p*‐value nodes being 100× smaller than the lowest ones

To characterize the developmental role of our candidate genes, we generated RNA‐Seq gene expression data for a developmental series of terminal leaves from a reference genotype. We refer to this dataset as LeafDev. Developmental time accounted for the largest proportion of variance in the LeafDev gene expression data. The apical region sample point was distinctly separated from later developmental stages, and there was more extensive separation of later stages (Figure 5a). We used these data to perform differential expression analysis (Figure 5b) and to confirm that we observed the expected expression profiles for Gene Ontology (GO) categories (Figure 5c) and homologs of known leaf development regulators (Figure 5d). To be able to examine the relationship with network topology of our GWAS and LeafPop DEG candidate genes, we performed unsupervised network analysis to obtain an unbiased overview of major expression profiles and underlying processes active during leaf development and to identify the most central genes involved in these processes. We calculated a gene co‐expression network (Table 2) by aggregating networks from multiple inference algorithms (Marbach et al., 2012). Our analysis involved graph partitioning to define modules (clusters) of genes and subsequent calculation of node centrality statistics (Table 3, Table S4). We identified statistical enrichment of GO and PFAM (protein family) categories within graph clusters to annotate common processes represented by cluster members (Figure S5, Table S5). We overlaid stage‐wise DE results (Table S6) onto the network nodes to visualize network regions or clusters active at each leaf development stage transition and to examine whether most significantly DE genes (DEGs) exhibited an increase or decrease in expression (Figure 6).

**TABLE 2:** Summary statistics for the LeafDev gene co‐expression network

Statistic	LeafDev network
Number of nodes	14,419
Number of edges	160,414
Connected components	325
Global clustering coefficient	0.293769
Scale‐free fit	0.869421
Average degree	22.2504
Average weighted degree	6.95192
Network diameter (largest component)	10.0627
Average path length	1.31698

**TABLE 3:** Genes with high gene co‐expression network centrality measures in the LeafDev co‐expression network. Centrality measures are PageRank (PR), betweenness centrality (BT), eigenvector centrality (EV), Katz centrality (KT), and Kleinberg's authority (AT). The top five genes for each network metric are detailed. Gene descriptions and best *Arabidopsis thaliana* BLASTp hits were obtained from PlantGenIE. *A. thaliana* homologs with reported leaf or whole plant phenotypes in the RARGE (RIKEN Arabidopsis Genome Encyclopedia) database are indicated in bold

Gene	Description	ATG homolog	PR	BT	EV	KT	AT
Potra000252g00987	ADP‐ribosylation factor	AT1G10630				x	
Potra000353g01326	Histidine kinase 3	AT1G27320		x			
Potra000353g01329	Binding transcription activator	AT1G67310		x			
Potra000380g01682	Ribosomal protein S27	AT3G61110		x			
Potra000389g01777	Homeobox protein knotted (KNAT6)	AT1G23380	x				
Potra000404g01918	ATP synthase subunit epsilon, mitochondrial	AT1G51650			x		x
Potra001053g08992	Probable linoleate 9S‐lipoxygenase	AT1G55020		x			
Potra001073g09248	Acid‐amido synthetase GH3.6	AT5G54510	x				
Potra001485g12400	Homeobox protein knotted (KNAT1)	AT4G08150	x				
Potra001534g12731	Diphosphooligosaccharide‐protein glycosyltransferase subunit 4A	AT3G12587			x		
Potra001842g14793	Ubiquitin‐conjugating enzyme	AT1G78870			x	x	x
Potra002003g15717	Homeobox protein STM	AT1G62360	x				
Potra003440g21673	Actin‐depolymerizing factor	AT5G59880				x	
Potra003719g22556	Macrophage migration inhibitory factor	AT5G01650			x	x	x
Potra003830g23027	Alpha type‐7	AT5G66140			x	x	x
Potra004406g24892	RNA‐binding protein	AT1G51510		x			
Potra006771g25788	NADH dehydrogenase [ubiquinone] 1 alpha subcomplex subunit	AT5G47890					x
Potra008191g26111	Glycosyl hydrolase superfamily protein	AT4G16260	x				

To aid community utilization, we have made the gene expression data and the co‐expression network available within PlantGenIE (Sundell et al., 2015) as the dataset "LeafDev."

### 3.4 Developmental characterization of population candidate genes

The majority of the GWAS candidate genes were expressed within the sampled LeafDev leaf ages but were not included in the developmental network, indicating low levels of connectivity (436 GWAS leaf area genes, 451 circularity genes, and 424 indent depth genes were included in the network). None of the candidate genes that were present in the LeafDev network were highly ranked in terms of network connectivity metrics, and none of the network clusters were enriched for these GWAS genes. Furthermore, we performed gene set enrichment analysis (GSEA) of the GWAS genes using network PageRank score as a measure of network centrality to rank‐order genes within the LeafDev network, finding no significant enrichment (Daub et al., 2013). However, the trend was for GWAS genes being present among low connectivity genes (Figure S6). As such, genes identified by GWAS were clearly not central within the LeafDev network.

We performed GO enrichment analysis of the GWAS candidate genes; however, none had significant enrichment, suggesting that they included genes spanning a diverse range of biological processes and that no particular process was associated with the genetic control of trait variation among these genes. As an alternative to analyzing the discrete set of GWAS candidate genes, we used the SNP with the strongest association (lowest *p*‐value) within each gene to rank order *all* genes for each trait. We then performed GSEA using gene sets from GO terms and expression network clusters. A number of GO terms were enriched for the three traits, including “protein phosphorylation” (GO:0006468) for circularity, “sulfur compound metabolic process” (GO:0006790) for indent depth, and “carbohydrate metabolic process” (GO:0005975) for area. Among the network clusters, five were significant at a 5% false discovery rate. Clusters [1:4], [1:5], and [1:10] were enriched in the leaf area GWAS results, and clusters [1:1], [1:4], and [1:5] were enriched for circularity. No cluster was significant for indent depth.

**FIGURE 7:**
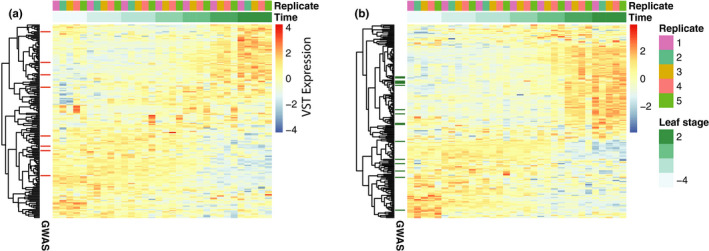
Developmental expression of genes differentially expressed between phenotypic extremes. The heat map shows the expression of the differentially expressed genes depicted in Figure 4. Expression in the terminal leaf development dataset is represented for genes differentially expressed between phenotypically extreme genotype sets for circularity (a) and indent depth (b). Genes that were found among the top 1,000 GWAS genes are indicated by colored lines on the left side of the heat map. Genes are clustered hierarchical clustering using average linkage. Samples are arranged by leaf age. Values depicted are VST (variance‐stabilizing transformation) normalized expression. Clustering was performed using complete linkage and correlation‐based distance

Many of the LeafPop DEGs between leaf shape extremes were actively regulated in the LeafDev dataset (Figure 7; Figure S7). There was no significant enrichment of GO terms among these sets of LeafPop DEGs; however, GSEA showed that indent depth GWAS genes were significantly enriched in indent depth DEGs (*q* = 0.0009), that circularity GWAS genes were significantly enriched in circularity DEGs (*q* = 0.009), but that there was no enrichment for area. Examination of the correlations between DEGs and the corresponding phenotypic traits showed that, although no single correlation was significant, correlation values for the DEGs were significantly higher than for non‐DEGs (Figure S4b). Two of these DEGs were in common for all three traits (Figure S8) but had relatively low and consistent expression within the LeafDev dataset: Potra196739g30199 (ATP synthase subunit C) and Potra003791g32371 (mRNA cap guanine‐N7 methyltransferase). Within the LeafDev network, 71 area, 83 circularity, and 85 indent depth DEGs were present. We performed GSEA of the sets of LeafPop DEGs within the LeafDev network (Figure S6), which revealed significant under‐representation for genes with low connectivity for all three traits (*p* = 0.004, 0.011, and 0.001, respectively).

The LeafPop data were previously utilized to perform expression quantitative trait locus (eQTL) mapping and co‐expression network analyses (Mähler et al., 2017). A relatively large proportion of the LeafPop DEGs were also eGenes (*i.e.,* genes for which an eQTL was present): 80 for area, 78 for circularity, and 83 for indent depth. Of these genes, 22 for circularity and 35 for indent depth were also part of the subset of genes that contributed to the GWAS GSEA signal. We examined the distribution of network centrality (degree) of DEGs and non‐DEGs and GWAS genes within the LeafPop co‐expression network presented in Mähler et al. (2017) and the LeafDev network (Figure S9) and performed GSEA to test for a relationship with network connectivity within the LeafPop co‐expression network (Figure S10). LeafPop DEGs had significantly lower centrality than non‐DEGs, a pattern that we confirmed was specific only to DEGs and not to random gene sets (Figure S9). The GSEA revealed that GWAS genes were significantly enriched among low connectivity genes, while DEGs were not, although the trend for DEGs was similar (Figure S10). Furthermore, DEGs displayed signatures of relaxed selective constraint (*i.e.,* lower negative selection), as indicated by Tajima's D values (Figure S11). This pattern was observed for DEGs but not for the top‐ranked 1,000 GWAS gene sets.

## 4 DISCUSSION

Despite the vast variation in leaf shape within and between species, we still have a rather limited understanding of the gene regulatory network underlying the process of leaf development and of the genetic determinants of leaf shape variation among individuals. In our current study system, there is extensive variation in leaf form within an individual (Figure 1a,b) as well as shape variation of pre‐formed leaves among individuals (Figure 2a). We used leaf area, circularity, and indent depth as representative physiognomy traits, with leaf shape being under tight genetic control (Table 1). There was no apparent link between leaf shape or size and environment, longitude, or latitude (Table 1) and *Q*_ST_ values were low. As such, there was no identifiable adaptive role or clear signature of directional selection for leaf shape within the SwAsp collection. In line with this, and in stark contrast to the date of autumn bud set, which is highly adaptive (Wang et al., 2018), we did not identify any significant SNP associations for the three traits. Given the high *H* ^2^ for leaf circularity and indent depth, this indicates complex genetic architecture with a large number of small‐effect size polymorphisms contributing to the control of leaf shape variation. As the population size of the SwAsp collection is certainly underpowered to detect such small‐effect associations, we reasoned that calculated *p*‐values would still be informative for rank ordering the importance of SNP associations. We therefore used this rank ordering to identify the top 1,000 associated genes (i.e., GWAS candidate genes) and examined these further. After normalizing for feature length, the highest density of SNPs within the candidate genes was observed in UTR and regulatory (up‐ and downstream) regions (Figure 3), suggesting that those SNPs potentially act by influencing gene expression.

We note that these findings are highly concordant with the omnigenic model (Boyle et al., 2017; Liu, Li, & Pritchard, 2019), which posits that a majority of genes contributing to trait variance are not directly biologically connected with the trait of interest (Liu et al., 2019). The model states that there is a limited set of genes that directly affect a trait (referred to as core genes), while all other genes with expression variation in the relevant tissue are peripheral to the trait. These peripheral genes can have *trans*‐acting effects that propagate through the highly connected regulatory network that cause individually small changes in the expression of core genes. While each *trans*‐effect is small in isolation, the combined effect of these peripheral *trans*‐acting effects will explain the majority of the variation in core genes and, therefore, in trait variation. It has not been resolved whether core genes defined on the basis of topology within a co‐expression network represent core genes as defined by the omnigenic model. It also remains to be explored whether the ability to predict phenotype from gene expression profiles, or co‐expression modules, is an effective means of identifying omnigenic core genes.

We further explored network topology of our identified GWAS candidate genes. A common characteristic of core genes (as defined by the omnigenic model) and of network hubs is that they are under strong selective constraint as natural selection acts strongly against large‐effect variants (Gouy, Daub, & Excoffier, 2017; Liu et al., 2019; Simons, Bullaughey, Hudson, & Sella, 2018). To test whether this held up in our system, we focused on a reference genotype to establish a developmental timeline for terminal leaves. As predicted by the model, our GWAS candidate genes were not hubs within the LeafDev co‐expression network.

Our observation that the highest density of top‐ranked SNPs was within regulatory regions prompted us to make use of an existing resource of gene expression data (LeafPop) assaying flushing buds from the same population (Mähler et al., 2017). We have previously performed eQTL mapping using these data, the results of which also highlighted the importance of SNPs located within regulatory regions in controlling natural variation in transcript abundance (Mähler et al., 2017). We did not identify any genes with significant correlation of gene expression variation among genotypes to variation in our target leaf traits, and correlations were of low magnitude (Figure S4). This is, however, expected given the apparently highly polygenic nature of these traits.

In addition to the GWAS candidate genes, we also derived a set of candidate genes based on the LeafPop expression data. To this end, we identified a set of genes with the maximum signal strength between expression and phenotype by performing DE analyses between sets of genotypes with the most extreme phenotype values (Figure 4). There was significantly higher correlation for DEGs than non‐DEGs and three‐way overlap between GWAS, DEGs, and eQTL presence provided circumstantial evidence that these genes may influence leaf shape via gene expression variation and that this effect is under genetic control. The sets of DEGs were of lower centrality within the LeafPop co‐expression network (Figure S10) and displayed evidence of weaker negative selection (Figure S11). However, for area and indent depth there were fewer low connectivity genes within the LeafDev co‐expression network than expected by chance. As such, these candidate genes were under‐represented for genes for which changes to expression would be expected to have the lowest impact to the network in general and, by extension, on phenotype. The majority of these genes have no known function in leaf development, but many were actively regulated during leaf development (Figure 7, Figure S7). These results are highly concordant with the omnigenic model, indicating that the DEG analysis potentially identified a set of core trait genes (as defined by the omnigenic model) that have higher effect size on phenotype and higher correlation of expression to phenotype alongside a larger set of genes that are peripheral to the phenotype and that are distributed throughout the developmental co‐expression network. Within the GWAS candidate genes, there was no such under‐representation, suggesting that the DEGs were enriched for genes of higher importance within the development co‐expression network and, therefore, of larger impact on phenotype, although this signal is weak. The lack of GO enrichment within the GWAS or DEG candidate gene sets is also congruent with the omnigenic model as the majority of genes contributing to trait heritability are expected to be peripheral and not associated with particular biological processes or directly connected to the focal trait. Taken together, our results suggest that leaf shape is an omnigenic trait, with variation resulting from SNPs within regulatory regions that act by causing variation in gene expression. The genes affected are enriched within the periphery of the population co‐expression network and are associated with signatures of relaxed selective constraint. There was a lack of evidence for directional selection acting on leaf shape, as indicated by low subpopulation differentiation and a lack of any link to environmental variables. This potentially indicates that variation in leaf shape is adaptively neutral within the morphospace represented among the sampled genotypes, although there are other plausible explanations congruent with the results. However, neutrality does appear to be a harmonious interpretation as allele frequencies of SNPs among the GWAS candidate genes reflected those of all SNPs globally and of the 1,000 lowest ranked SNPs. There was, therefore, no clear indication of balancing or stabilizing selection or of extensive purifying selection. Of note, the pattern was not the same for area, for which SNPs within GWAS candidate genes showed a skew toward lower allele frequencies. This suggests that there may be contrasting selection pressures acting on leaf size and shape, with leaf size being subjected to stronger purifying selection, although the signal was weak, and this was not reflected in the distribution of Tajima's D (Figure S11).

Similar approaches have been used to identify and prioritize candidate genes in maize, with small numbers of those candidates confirmed to influence leaf characteristics in transgenic lines (Baute et al., 2015, 2016; Schaefer et al., 2018). A distinct difference in maize is the clear separation of cell proliferation and subsequent expansion into defined zones that can easily be sampled separately. This sampling approach increases signal strength for associating gene expression to division and expansion processes, which were mixed within single samples within our current study. In maize, there are reported loci of higher effect size influencing leaf development and phenotype, possibly as those traits are correlated with general yield characteristics (Baute et al., 2016) and have therefore been targets of artificial selection. A similar study in apple reported a small number of significant associations for leaf shape traits but concluded that those were false positives (Crouch et al., 2018) and a study in sweet potato used a similar DEG approach to identify genes associated with variation in leaf form (Gupta et al., 2019).

The analysis of leaf shape has many parallels with that of face shape in humans, for which initial GWAS of basic parameters (such as length:width) yielded few to no significant associations. However, analyses that defined more specific subfeatures have yielded substantially more informative associations (Crouch et al., 2018). In aspen, there is similarly considerable scope to improve phenotype GWAS results through improved software tools to deconvolute more specific features of leaf shape, such as the angle of veins or of specific regions of the leaf, such as the base and tip. Each of these features is likely under specific molecular control that exhibits genetic variation among individuals and that are not captured by the larger‐scale features, such as overall leaf circularity, employed in our current study. The fact that such subfeatures of leaf shape are under local, spatially defined control and that growth distribution across the leaf lamina controls final leaf shape and venation patterning (Runions, Tsiantis, & Prusinkiewicz, 2017) may also explain the low correlation between leaf shape traits and gene expression in the whole‐bud samples used to generate the gene expression values for the eQTL mapping results that we considered here. Furthermore, the use of a single developmental snapshot for such analysis is limiting as causal variation in expression could occur at any point during the developmental program.

We integrated developmental gene expression profiling, GWAS, population‐wide gene expression data, and population genetics to define the genetic architecture of leaf shape variation in aspen. We show that leaf shape variation is a highly complex trait likely determined by small‐effect variations in gene expression caused by numerous small‐effect size SNPs in regulatory regions. Genes with evidence of association with variation in leaf shape were peripheral within the population‐wide gene co‐expression network, were not hubs within the leaf developmental network, and displayed signatures of relaxed selection. We therefore suggest that leaf shape is an omnigenic trait. Combined with low subpopulation differentiation and a lack of correlation to climatic or environmental variables, we suggest that variation in leaf shape within the morphospace represented within the SwAsp collection may be neutral. We believe that this integrated approach is a pragmatic strategy for identifying candidate genes and disentangling the genetic basis of complex traits, such as leaf shape variation in aspen, which have no apparent evidence of being adaptive and for which no significant associations are identified using association mapping.

## CONFLICT OF INTEREST

The authors have no conflicts of interest to declare.

## AUTHOR CONTRIBUTION

**Niklas Mähler:** Data curation (equal); Formal analysis (lead); Investigation (equal); Validation (equal); Visualization (equal); Writing‐original draft (supporting); Writing‐review & editing (supporting). **Bastian Schiffthaler:** Data curation (equal); Formal analysis (equal); Investigation (equal); Methodology (equal); Software (lead); Visualization (equal); Writing‐original draft (supporting); Writing‐review & editing (supporting). **Kathryn M. Robinson:** Conceptualization (equal); Data curation (equal); Formal analysis (equal); Investigation (equal); Visualization (supporting); Writing‐review & editing (supporting). **Barbara Terebieniec:** Formal analysis (supporting); Investigation (supporting); Writing‐review & editing (supporting). **Matej Vucak:** Conceptualization (supporting); Methodology (supporting). **Chanaka Mannapperuma:** Data curation (equal); Software (equal); Visualization (equal). **Mark Bailey:** Methodology (supporting); Supervision (supporting); Writing‐original draft (supporting); Writing‐review & editing (supporting). **Stefan Jansson:** Funding acquisition (supporting); Writing‐review & editing (supporting). **Torgeir R R Hvidsten:** Methodology (supporting); Writing‐original draft (supporting); Writing‐review & editing (supporting). **Nathaniel Street:** Conceptualization (lead); Formal analysis (equal); Funding acquisition (lead); Investigation (equal); Methodology (equal); Project administration (lead); Resources (lead); Supervision (lead); Visualization (supporting); Writing‐original draft (lead); Writing‐review & editing (lead).

## AUTHOR CONTRIBUTIONS

NRS: Plan and design of the research. NM, BS, KMR, and BKT: Experiments. NRS and SJ: Contribution to resources. MV, MESB, and TRH: Contribution to analysis ideas. NM, BS, KMR, BKT, and CM: Data analysis. NRS with contributions from all authors: Writing of the manuscript.

## Supporting information

Fig S1

Fig S2

Fig S3

Fig S4

Fig S5

Fig S6

Fig S7

Fig S8

Fig S9

Fig S10

Fig S11

Table S1

Table S2

Table S3

Table S4

Table S5

## Data Availability

The LeadDev data are deposited in the European Nucleotide Archive (ENA) as accession PRJEB31491. Table S6 is available from the Dryad data repository at https://doi.org/10.5061/dryad.3n5tb2rdt.
