# Supplementary figures and images for: Leaf shape in Populus tremula is a complex, omnigenic trait
Source: Ecol Evol. 2020 Oct 13;10(21):11922–40. doi: 10.1002/ece3.6691 (PMC7663049; doi:10.1002/ece3.6691)

A

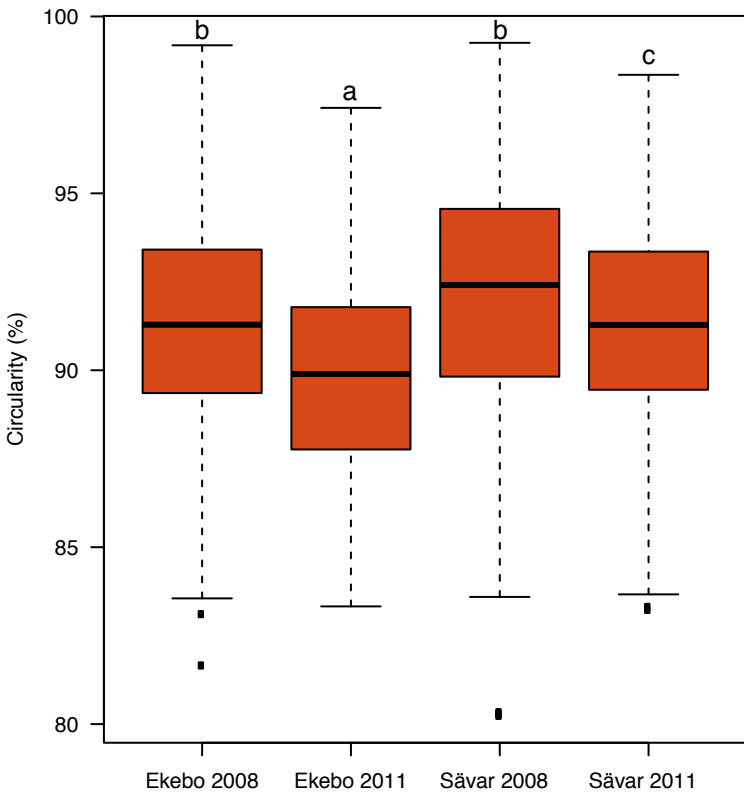

B

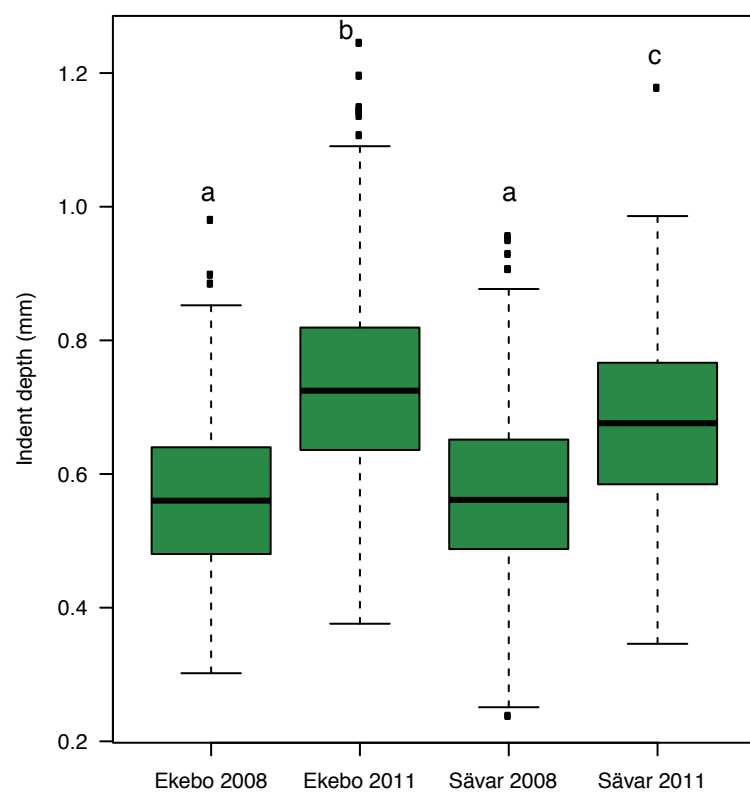

C

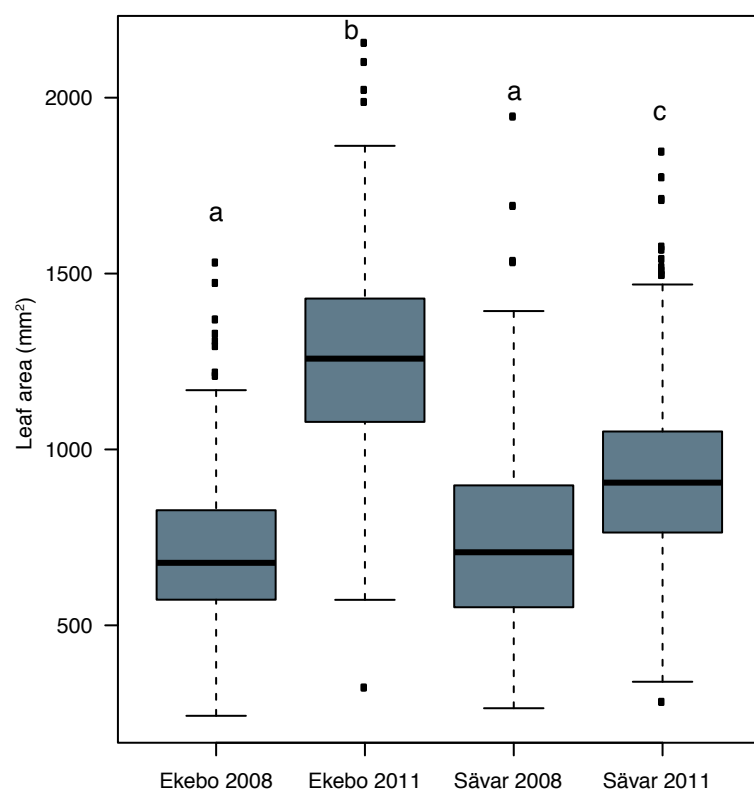

Supplement: Supplementary file 1 — Fig S1 [file ECE3-10-11922-s001.pdf]

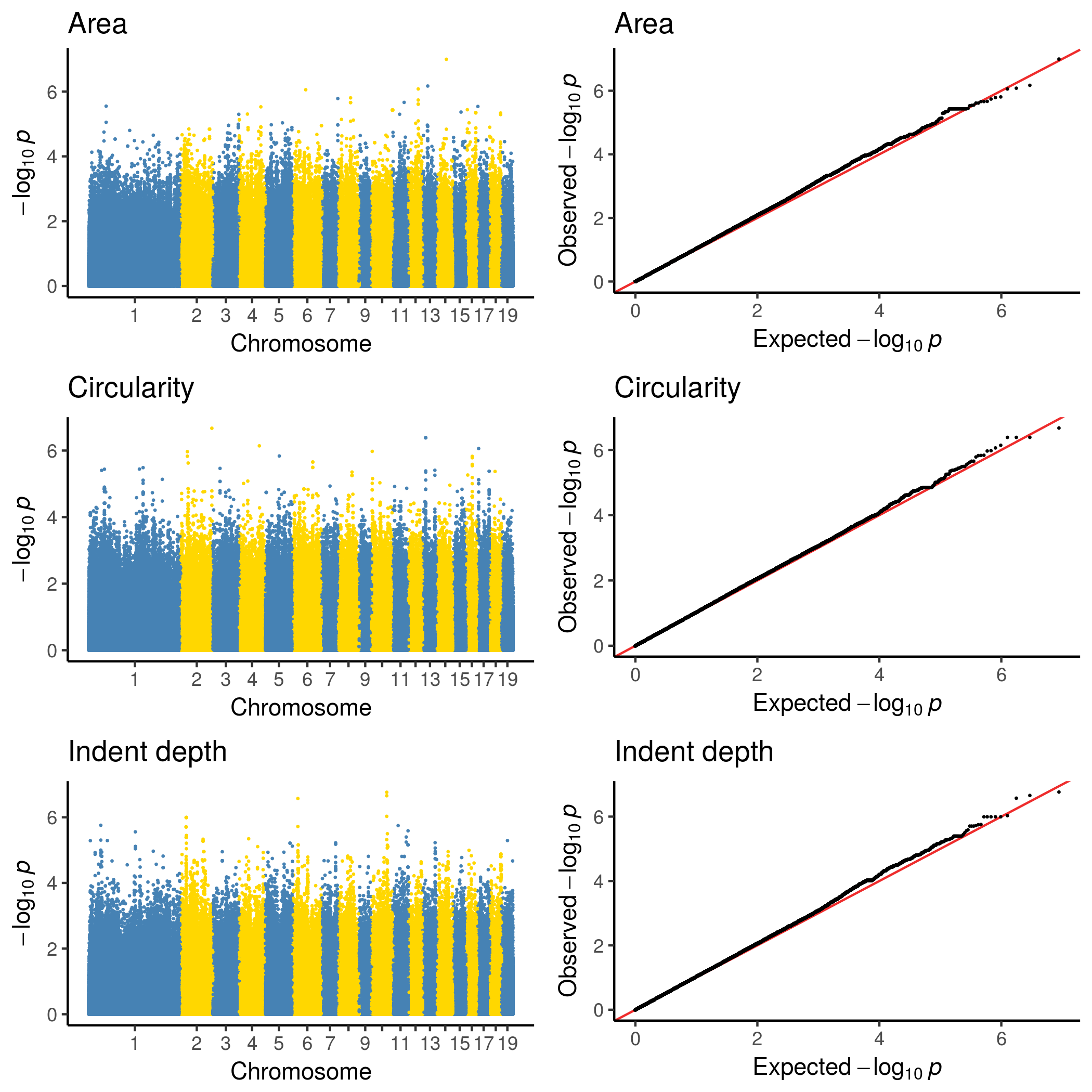

Supplement: Supplementary file 2 — Fig S2 [file ECE3-10-11922-s002.png]

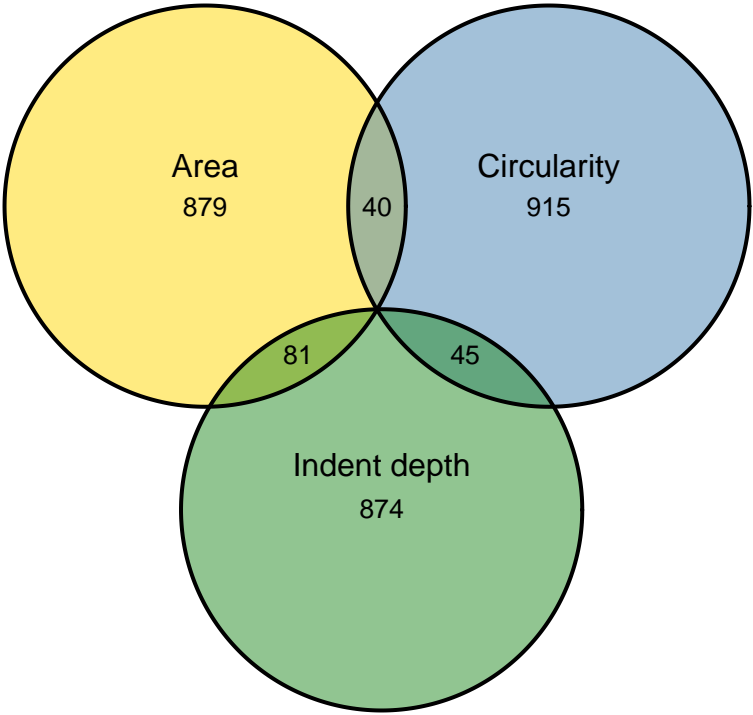

Supplement: Supplementary file 3 — Fig S3 [file ECE3-10-11922-s003.pdf]

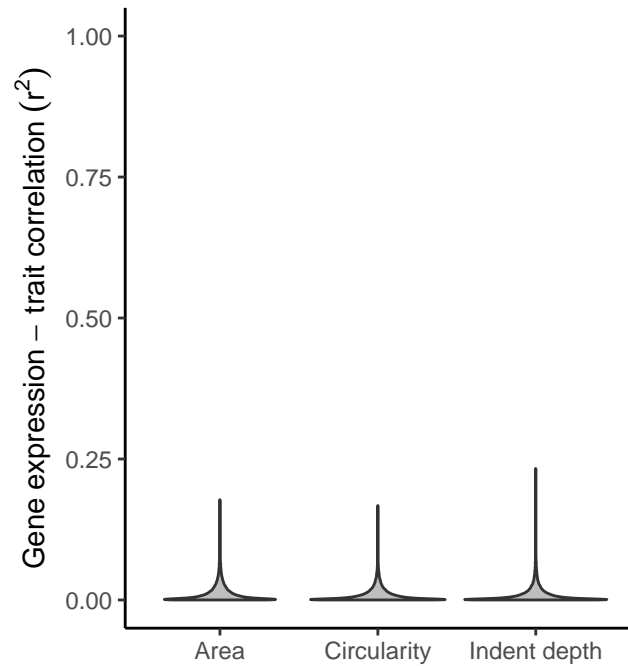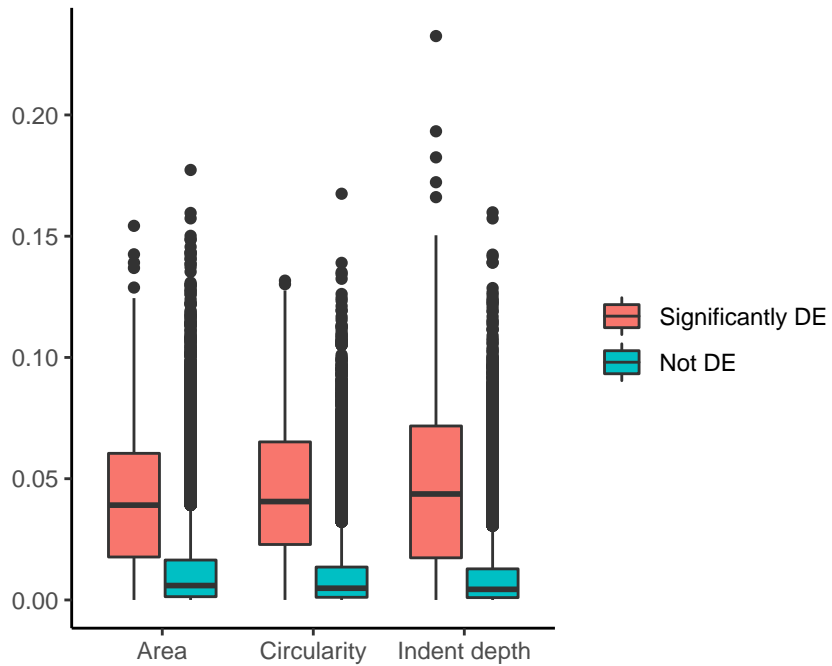

Supplement: Supplementary file 4 — Fig S4 [file ECE3-10-11922-s004.pdf]

## Area GWAS

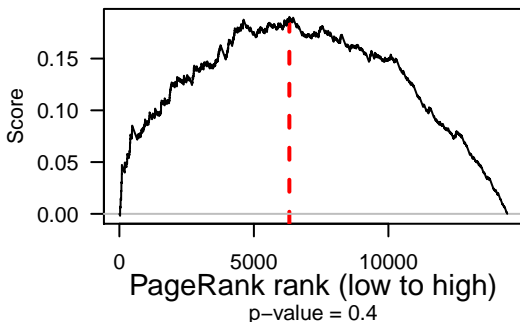

## Area DEGs

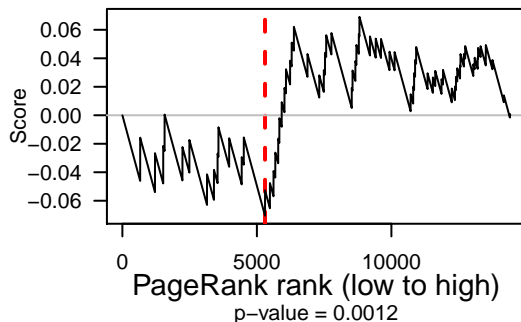

## Circularity GWAS

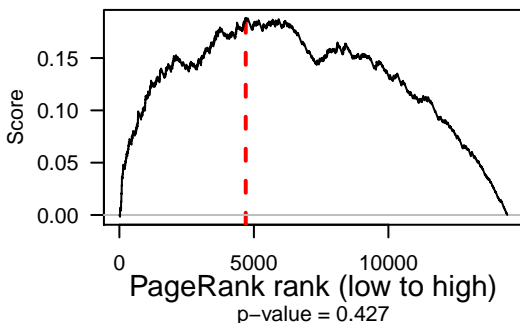

## Circularity DEGs

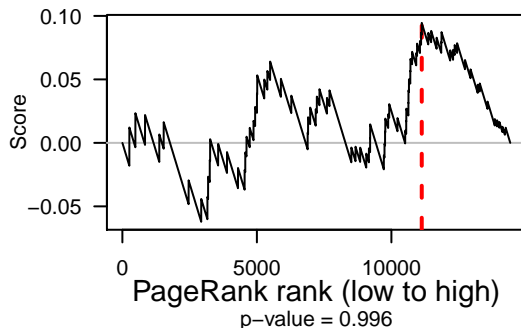

## Indent depth GWAS

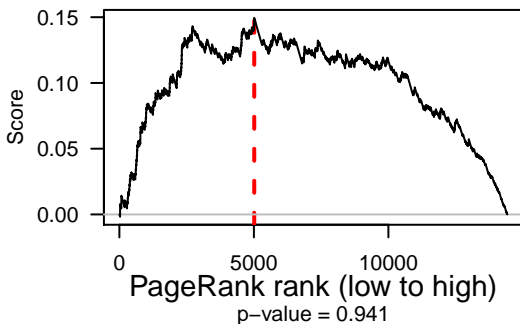

## Indent depth DEGs

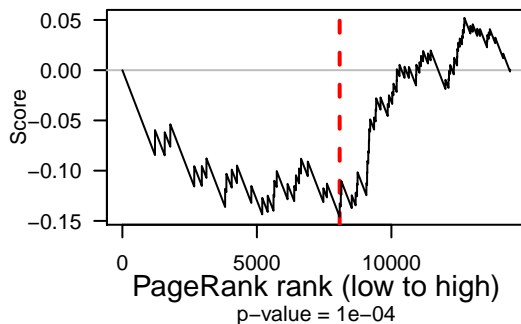

Supplement: Supplementary file 6 — Fig S6 [file ECE3-10-11922-s006.pdf]

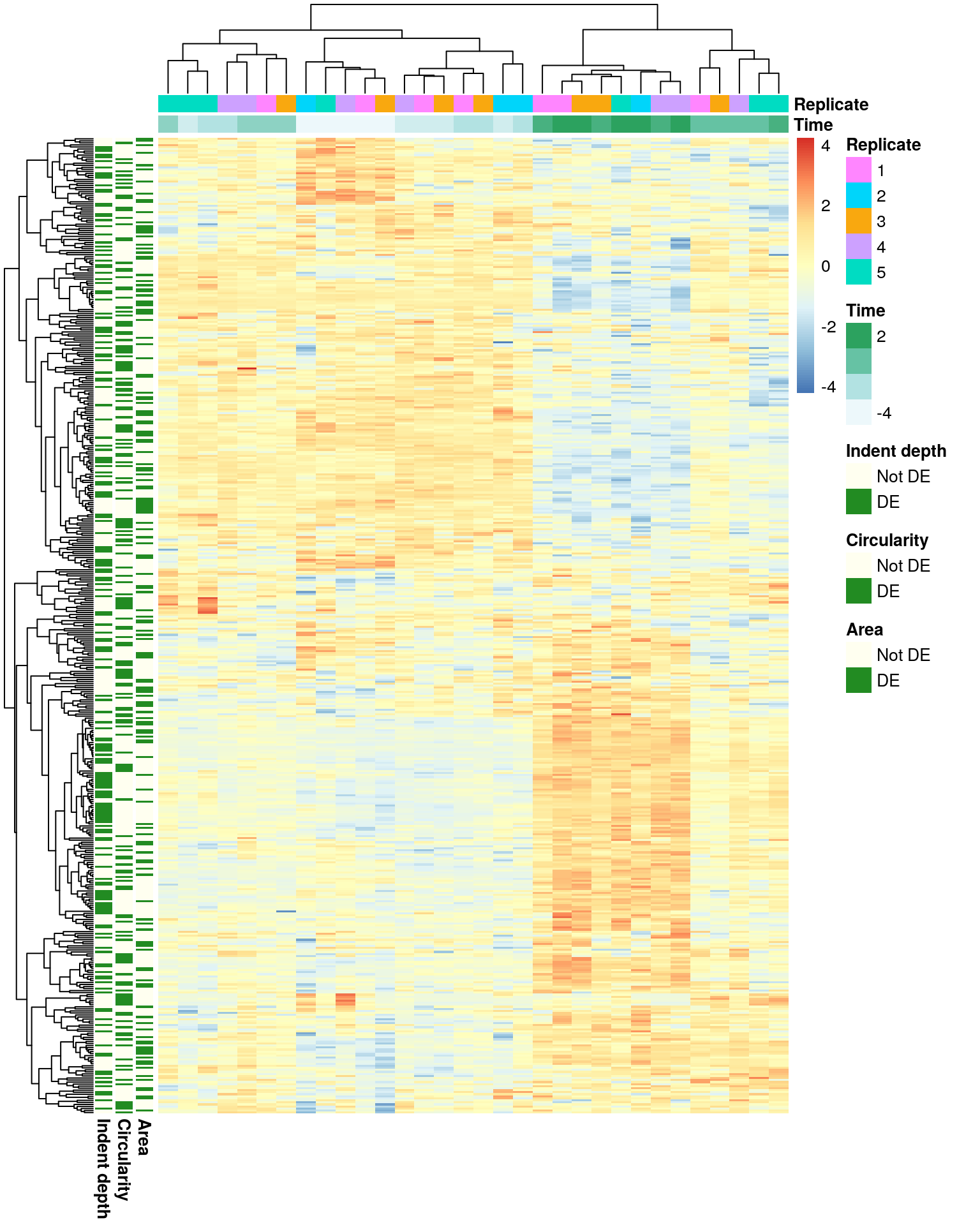

Supplement: Supplementary file 7 — Fig S7 [file ECE3-10-11922-s007.png]

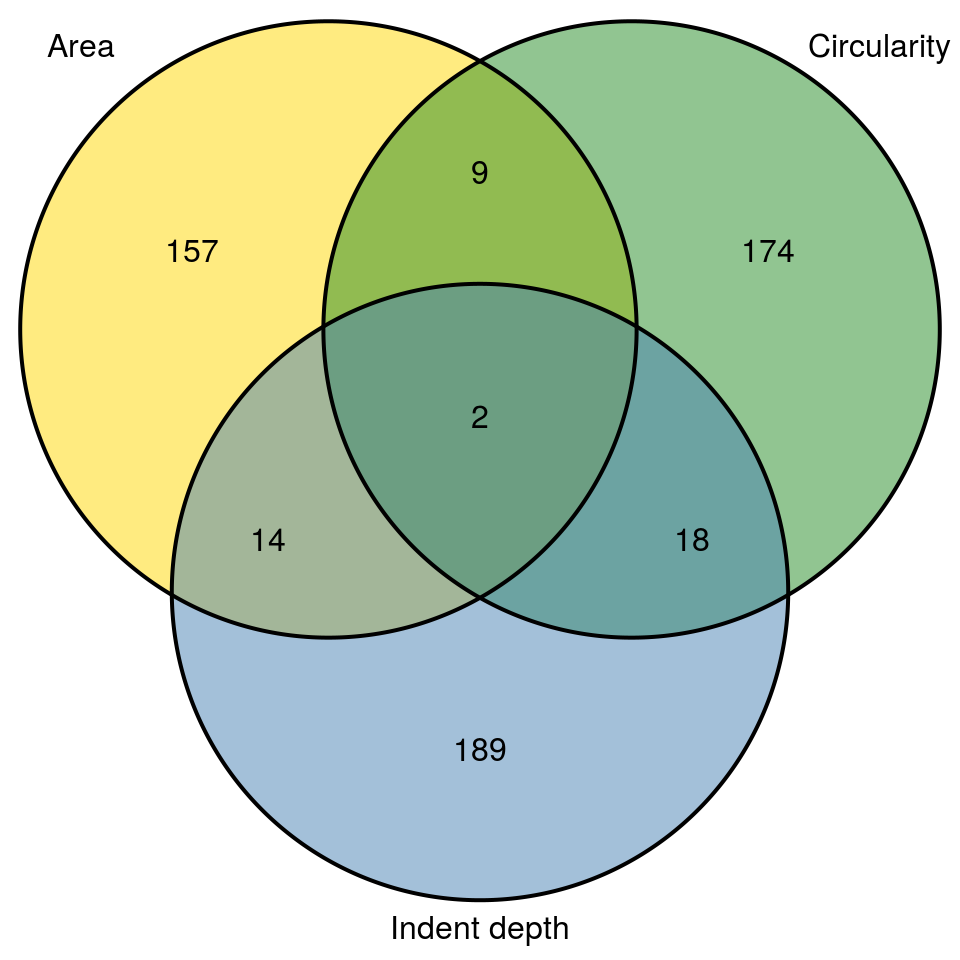

Supplement: Supplementary file 8 — Fig S8 [file ECE3-10-11922-s008.png]

Circularity

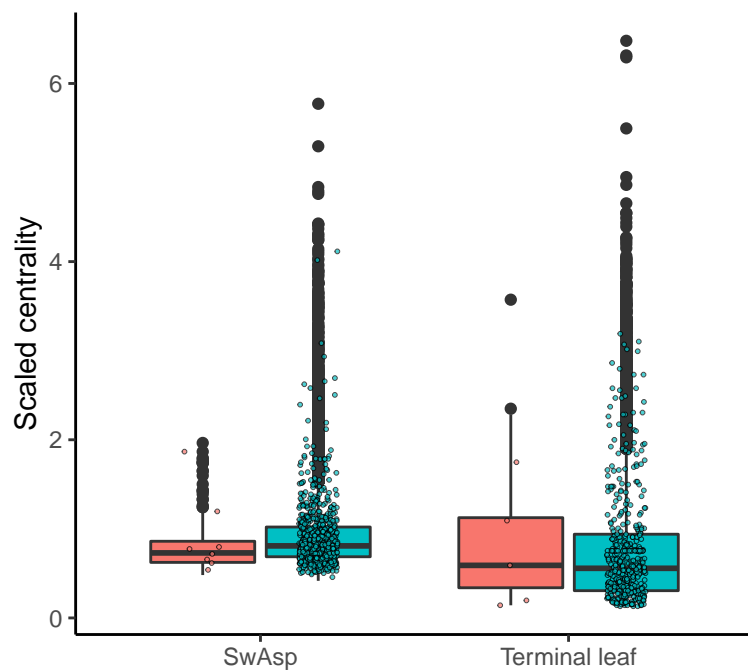

Indent depth

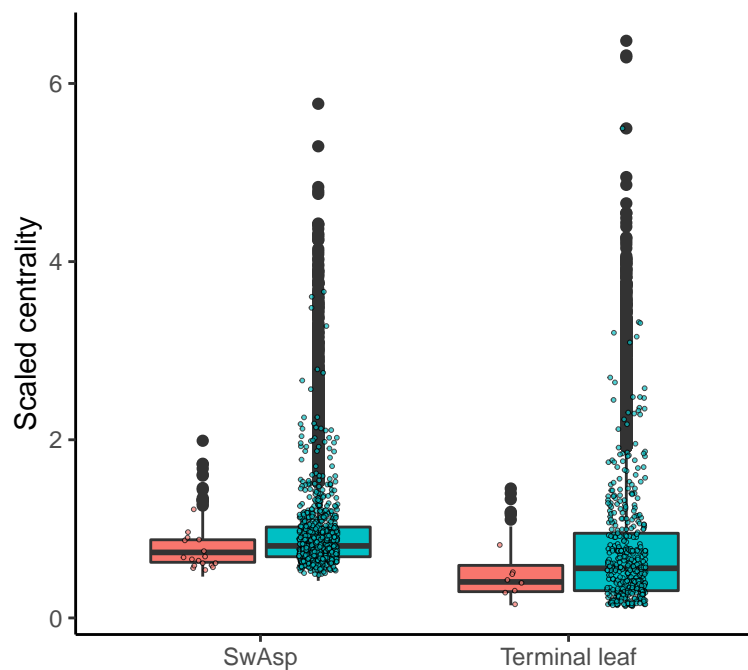

Area

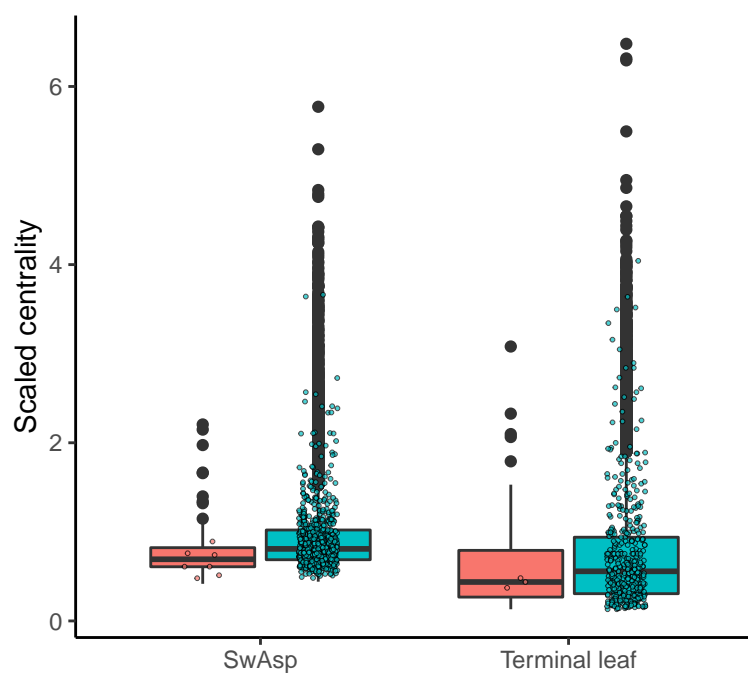

Random area

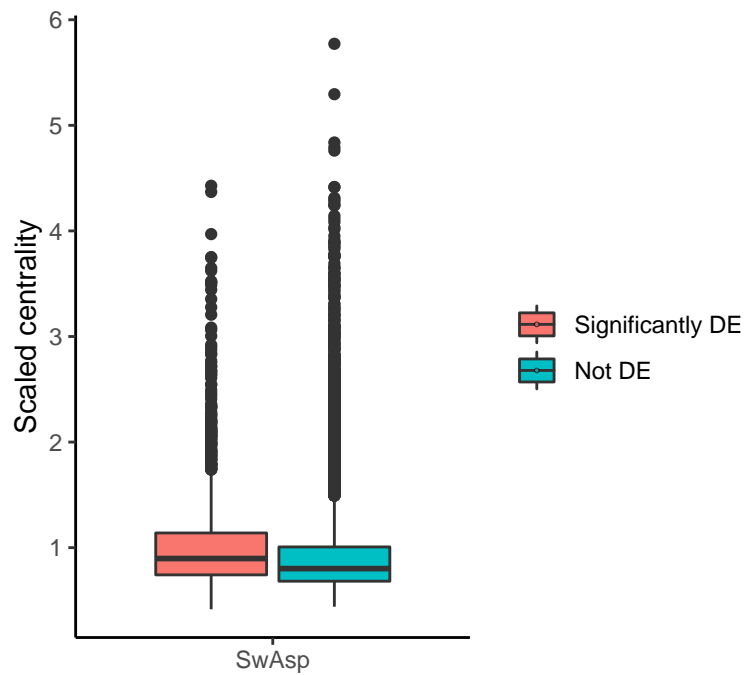

Supplement: Supplementary file 9 — Fig S9 [file ECE3-10-11922-s009.pdf]

## Area GWAS

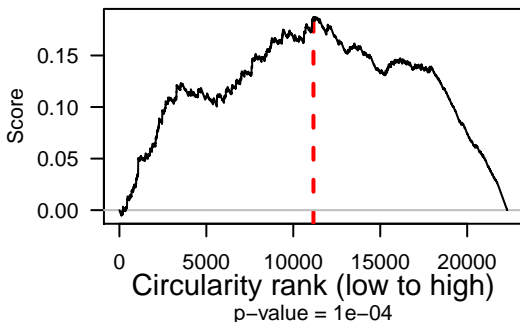

## Area DEGs

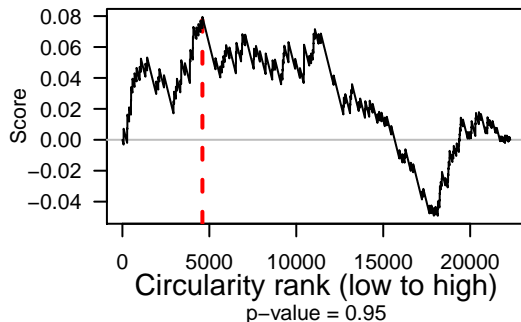

## Circularity GWAS

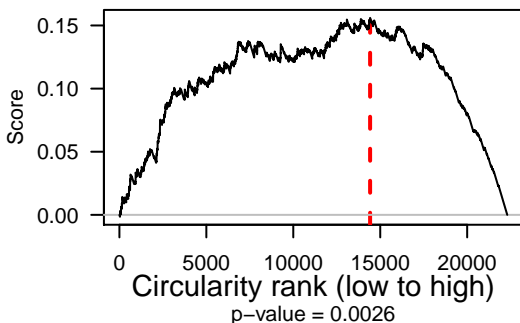

## Circularity DEGs

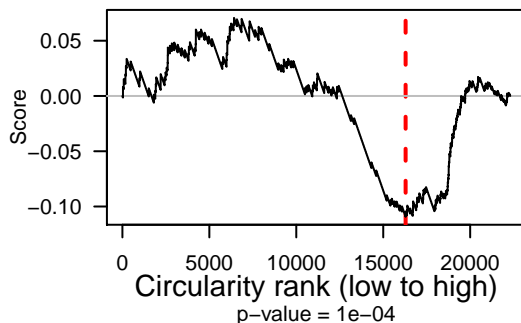

## Indent depth GWAS

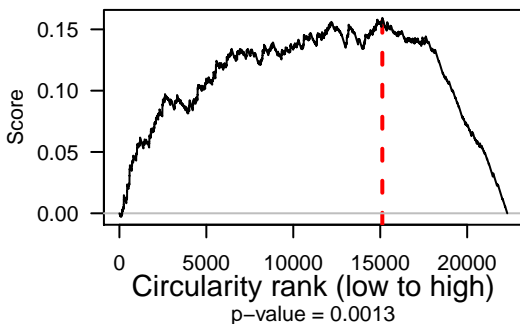

## Indent depth DEGs

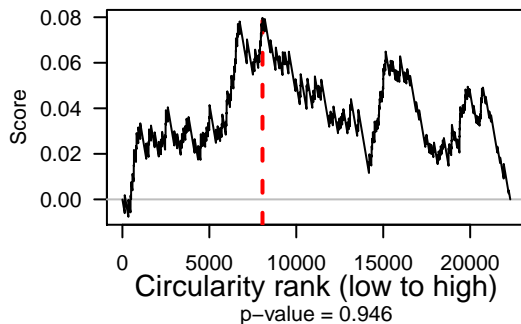

Supplement: Supplementary file 10 — Fig S10 [file ECE3-10-11922-s010.pdf]

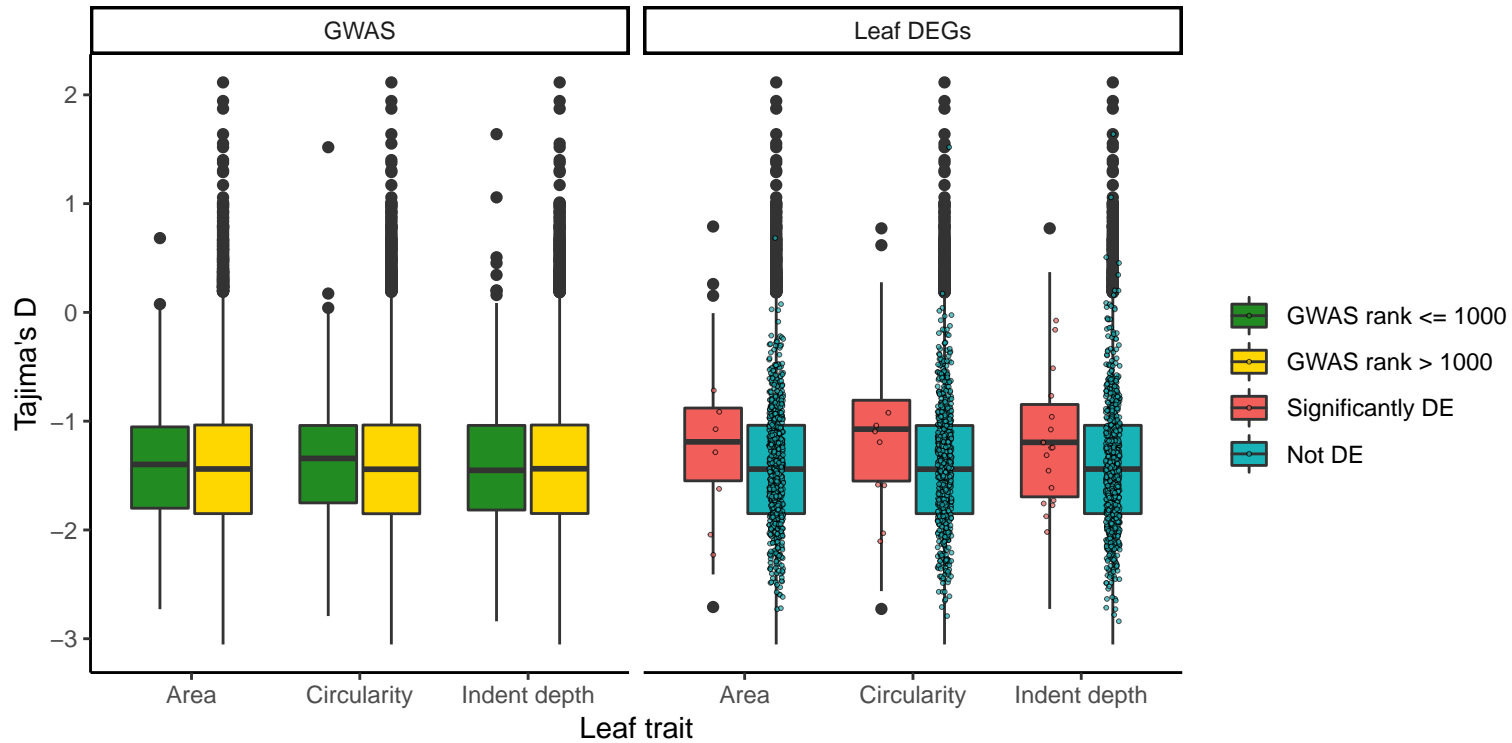

Supplement: Supplementary file 11 — Fig S11 [file ECE3-10-11922-s011.pdf]
