# Supplementary material for: Leaf shape in Populus tremula is a complex, omnigenic trait
Source: Ecol Evol. 2020 Oct 13;10(21):11922–40. doi: 10.1002/ece3.6691 (PMC7663049; doi:10.1002/ece3.6691)
Supplement: Supplementary file 5 — Fig S5 [file ECE3-10-11922-s005.pdf]

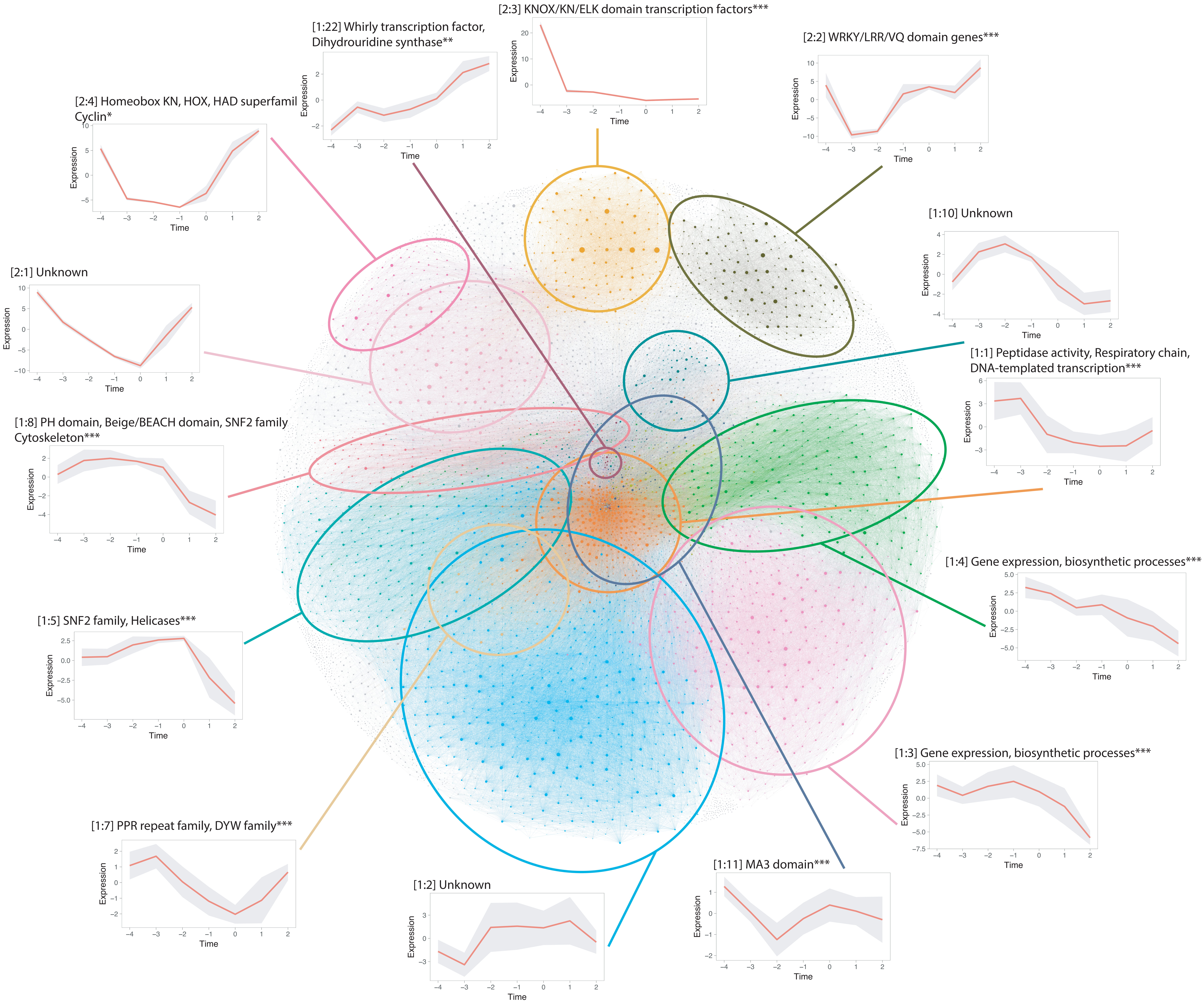

[2:3] KNOX/KN/ELK domain transcription factors***
[1:22] Whirly transcription factor, Dihydrouridine synthase**
[2:2] WRKY/LRR/VQ domain genes***
[2:4] Homeobox KN, HOX, HAD superfamil Cyclin*
[1:10] Unknown
[2:1] Unknown
[1:1] Peptidase activity, Respiratory chain, DNA-templated transcription***
[1:8] PH domain, Beige/BEACH domain, SNF2 family Cytoskeleton***
[1:4] Gene expression, biosynthetic processes***
[1:5] SNF2 family, Helicases***
[1:3] Gene expression, biosynthetic processes***
[1:7] PPR repeat family, DYW family***
[1:11] MA3 domain***
[1:2] Unknown
Expression
Time
